# GC–MS/MS analysis of seminal plasma PUFAs in distinct subgroups of infertile men: diagnostic potential and insight into mechanisms of male infertility

**DOI:** 10.1038/s41598-025-18044-4

**Published:** 2025-10-03

**Authors:** Kamil Rodak, Magdalena Grajzer, Izabela Kokot, Ricardo Faundez, Iwona Gilowska, Anna Prescha, Ewa Maria Kratz

**Affiliations:** 1 Department of Laboratory Diagnostics, Division of Laboratory Diagnostics, Faculty of Pharmacy, Wroclaw Medical University, Borowska Street 211A, 50-556 Wrocław, Poland; 2 Research and Development Center, INVICTA, Polna Street 64, 81-740 Sopot, Poland; 3 Department of Dietetics and Bromatology, Wroclaw Medical University, Borowska Street 211, 50-556 Wrocław, Poland; 4 InviMed Fertility Clinics, Rakowiecka Street 36, 02-532 Warsaw, Poland; 5 Institute of Health Sciences, Collegium Salutis Humanae, University of Opole, Katowicka Street 68, 45-060 Opole, Poland; 6 Reference Center for the Diagnosis and Treatment of Infertility, Clinical Center of Gynecology, Obstetrics and Neonatology in Opole, Reymonta Street 8, 45-066 Opole, Poland

**Keywords:** Male infertility, Seminal plasma, Polyunsaturated fatty acids (PUFAs), Gas chromatography–tandem mass spectrometry (GC–MS/MS), Biomarkers, Biochemistry, Biomarkers, Diseases, Medical research

## Abstract

Infertility affects approximately 17.5% of the adult population, with the male factor contributing to nearly half of the cases. Seminal plasma has emerged as a potential source of biomarkers and insights into the mechanisms of male infertility; however, the significance of polyunsaturated fatty acids (PUFAs) in this body fluid remains insufficiently explored. The main objectives of the present observational study were to compare selected PUFA (linoleic acid (LA), α-linolenic acid (ALA), γ-linolenic acid (GLA), arachidonic acid (AA), eicosapentaenoic acid (EPA), and docosahexaenoic acid (DHA)) concentrations and ratios in seminal plasma between fertile (n = 22, aged 21–47) and infertile (n = 250, aged 24–45) men, as well as within infertile subgroups, including idiopathic, to investigate their clinical utility and correlations with semen parameters. An optimized extraction procedure and a validated gas chromatography–tandem mass spectrometry (GC–MS/MS) method were employed to determine the concentrations of PUFAs in seminal plasma. Nonparametric tests, including Mann–Whitney U test, ANOVA rank test followed by Tukey’s post hoc test, Spearman’s rank test, and ROC curves were used for analyses. Among the twenty-one examined parameters, nine (LA, GLA, EPA, DHA, ALA/LA, LA/AA, EPA/ALA, EPA/GLA, EPA/AA) exhibited significant differences between fertile control group and infertile patients (0.493 vs. 0.762 μg/mL, *p* = 0.007; 0.029 vs. 0.035 μg/mL, *p* = 0.005; 0.007 vs. 0.009 μg/mL, *p* < 0.001; 1.519 vs. 2.174 μg/mL, *p* = 0.029; 0.066 vs. 0.053, *p* = 0.042; 0.248 vs. 0.305, *p* = 0.002; 0.168 vs. 0.216, *p* = 0.010; 0.230 vs. 0.283, *p* = 0.013; 0.003 vs. 0.004* p* = 0.045, respectively), with LA, GLA, LA/AA, EPA/ALA, and EPA/GLA demonstrating limited (AUC < 0.650), and EPA exhibiting moderate diagnostic value (AUC = 0.709). Among the subgroups of infertile patients, the teratozoospermic group displayed the most pronounced alterations in both individual PUFA concentrations and PUFA ratios compared to other groups (*p* < 0.05). Notably, DHA, LA/DHA, ALA/DHA, GLA/DHA, AA/DHA, EPA/DHA exhibited at least one moderate correlation with semen parameters (0.4 <|R|, *p* < 0.05). Despite its exploratory nature, the present study provides insights into the mechanisms underlying male infertility and their reflection in seminal plasma composition while simultaneously outlining new directions in identifying/excluding PUFA concentrations or ratios (especially LA, GLA, EPA, DHA, ALA/LA, LA/AA, EPA/ALA, EPA/GLA, EPA/AA) as potential biomarkers. Moreover, the use of GC–MS/MS method offers a novel approach for potential future diagnostic applications.

**Supplementary Information:**

The online version contains supplementary material available at 10.1038/s41598-025-18044-4.

## Introduction

Infertility is a malfunction of the female and/or male reproductive system, defined by the inability to achieve pregnancy after 12 months or more of regular, unprotected sexual intercourse^1^. Recent data from 2023 indicate that infertility affects approximately 17.5% of the adult population, with male factor contributing to reproductive failure in up to 50% of cases^2,3^. Over the past decades, a significant decline in semen quality among men has been observed, exerting detrimental consequences for reproductive health and global fertility rates^4–6^. Male infertility is a multifactorial condition resulting from genetic and non-genetic factors, including lifestyle factors such as diet with growing evidence highlighting the role of fatty acids in semen quality and male reproductive potential^7^. Furthermore, a growing body of evidence identifies oxidative stress as a key factor in the pathogenesis of male infertility, contributing to spermatozoa dysfunction through mechanisms such as lipid peroxidation, DNA fragmentation, and oxidative damage to proteins^7,8^. In fact, one of the most challenging and poorly understood conditions in reproductive medicine is idiopathic male infertility. Despite extensive diagnostic evaluations, approximately 30–40% of male infertility cases are classified as idiopathic, with no identifiable cause based on current clinical, hormonal, genetic, or imaging assessments^9,10^. Several potential risk factors have been implicated in idiopathic cases, including oxidative stress, subclinical hormonal imbalances, subtle genetic variants undetectable by routine testing, environmental exposures (such as pesticides and heavy metals), lifestyle factors like smoking and obesity, elevated scrotal temperature, hidden infections, and epigenetic alterations^9^. This high prevalence of idiopathic cases underscores the urgent need to propose novel biomarkers and deeper mechanistic insights to improve diagnosis, stratification, and personalized treatment strategies.

For men experiencing difficulties in achieving conception with their partners, available diagnostic tools remain limited, often relying solely on standard semen analysis, which can reveal abnormalities such as reduced sperm count (oligozoospermia), impaired motility (asthenozoospermia) and viability, altered sperm morphology (teratozoospermia) as well as mixed forms which can significantly reduce fertilization potential^11^. Although the above examination serves as a gold standard in the diagnostics of male infertility, it usually does not provide comprehensive insights into the underlying causes of this condition. Its limitations are particularly evident in cases of idiopathic and unexplained male infertility, where conventional semen parameters are within reference ranges despite impaired reproductive function^12,13^. Notwithstanding the significant advancements in medical science, the underlying causes of male infertility remain incompletely understood, making the elucidation of its mechanisms a subject of ongoing scientific investigation. Another challenge in the diagnosis of male infertility is the continuous revision of the World Health Organization (WHO) diagnostic criteria^11,14^. Consequently, there is a critical need to identify non-invasive advanced diagnostic methodologies and reliable biomarkers of male infertility that could enhance the diagnostic process, enable more precise patient stratification, and support clinicians in selecting optimal therapeutic and preventive strategies^15^.

In response to the growing prevalence of infertility, scientists’ attention has increasingly focused on seminal plasma, which role in the diagnostics of male infertility and in elucidating the mechanisms underlying its development has become more evident^16,17^. Seminal plasma provides essential energy substrates for spermatozoa and plays a crucial role in regulating their maturation, capacitation, and function within the female reproductive tract, thereby influencing their functional competence and fertilization potential^15,18^. The composition of this body fluid is highly diverse, containing many various bioactive molecules, including lipids and their metabolic derivatives^16,19^, and thus is a valuable source of biomarkers for non-invasive diagnostics of male fertility potential^12,16,19^. However, until now, seminal plasma has not been utilized commonly in diagnostics, partly due to the incomplete understanding of the diagnostic potential of its molecular components, and/or the highly time-consuming methods that require large volumes of this specific biological material^15^.

Polyunsaturated fatty acids (PUFAs) play an essential role in human homeostasis, serving both structural and metabolic functions as key components of phospholipid membranes, including spermatozoa, where they maintain membrane fluidity and functionality as well as precursors for bioactive lipid mediators such as prostaglandins, leukotrienes, and resolvins, which regulate inflammation, oxidative balance, and cellular signaling pathways^20,21^. Due to the lack of endogenous biosynthesis, PUFAs must be obtained through dietary intake^22^. The most physiologically relevant are omega-3 PUFAs such as α-linolenic acid (ALA, 18:3 n-3), eicosapentaenoic acid (EPA, 20:5 n-3), and docosahexaenoic acid (DHA, 22:6 n-3), as well as omega-6 PUFAs, including linoleic acid (LA, 18:2 n-6 *cis*), γ-linolenic acid (GLA, 18:3 n-6), and arachidonic acid (AA, 20:4 n-6)^21^. Previous research has confirmed the presence of PUFAs (especially DHA) in spermatozoa membranes, seminal plasma, and whole ejaculate, highlighting the fundamental role in male fertility^7,23–28^. As integral components of sperm cell membranes, PUFAs contribute to the maintenance of membrane fluidity, flexibility, stability, and integrity^7,29–31^. These properties are essential for key reproductive processes, including capacitation, the acrosome reaction, and sperm-oocyte fusion^31–34^. Also, it has been reported that sperm motility, morphology, and concentration are positively associated with PUFA levels and ratios^26,35^. Although omega-3 PUFAs exhibit antioxidative properties, the presence of multiple unsaturated bonds in the chemical structure of PUFAs renders them particularly susceptible to oxidative damage and lipid peroxidation, leading to sperm membrane damage^20,31,36^. Consequently, this process may result in reduced sperm motility, viability, and morphological abnormalities^21,28^. The release of PUFAs into the spermatozoa’s microenvironment—seminal plasma—may further influence sperm function i.e. through their metabolic derivatives^21,28^. PUFAs are enzymatically converted into bioactive eicosanoids, such as prostaglandins, leukotrienes, and thromboxanes, whereas non-enzymatic pathways lead to isoprostanes formation^20,21^. Omega-3-derived metabolites exert anti-inflammatory, vasodilatory, and anti-platelet aggregation effects, whereas omega-6 derivatives exhibit opposing properties, promoting inflammation, vasoconstriction, and platelet aggregation^20,37^.

Although the levels of PUFAs and PUFA ratios in spermatozoa membranes and whole ejaculate as well as their correlations with semen quality parameters have already been studied, their diagnostic and therapeutic potential, as well as the mechanisms underlying changes in their levels and biological activity in seminal plasma remains poorly understood^21,23,25–27,35^. This may be attributed not only to the complex composition of seminal plasma, which complicates the determination of the source and function of individual lipids, making it unclear whether PUFA levels in the seminal plasma directly influence spermatozoa function or merely reflect the overall metabolic and/or oxidative status of the organism but also to the lack of standardized quantification methods.

Taking into account all the above information and needs in male infertility/reduced fertility diagnostics, the present observational case–control study aimed to evaluate the differences in the concentrations of selected PUFAs (LA, ALA, GLA, AA, EPA, and DHA) as well as their ratios, in seminal plasmas derived from fertile and infertile men and to identify or exclude potential biomarkers of male infertility, including those with an idiopathic background. Moreover, the correlations between these lipid parameters and standard semen parameters (such as sperm concentration, total sperm count per ejaculate, viability, total and progressive motility, and morphology) were assessed. The results were interpreted in the context of the latest WHO guidelines for male infertility diagnosis published in 2021^1^. Additionally, the impact of the revised 2021 diagnostic criteria on infertility classification was investigated by comparing outcomes within the infertile men groups classified according to the WHO standards from 2010 and 2021^1,38^. For PUFAs profiling in human seminal plasma, a modified GC–MS/MS methodology was used. To the best of our knowledge, the present study is the first to successfully utilize GC–MS/MS for the analysis of PUFA concentrations in human seminal plasma.

## Materials and methods

### Study participants and sample collection

The seminal plasma samples from infertile (n = 250, aged 24–45) and fertile (n = 22, aged 21–47) men were collected in collaboration with the Clinical Center of Gynecology, Obstetrics, and Neonatology in Opole, Poland, as well as the InviMed Fertility Clinics in Warsaw and Wroclaw, Poland. All study participants signed an informed consent to participate in the study, and the study protocol was approved by the Bioethics Human Research Committee of Wroclaw Medical University (KB-739/2022, KB-201/2024 and KB-580/2024). All procedures for sample collection were conducted following the International Conference on Harmonization Good Clinical Practice (ICH-GCP) guidelines and the principles of the IInd Declaration of Helsinki.

The inclusion criteria for the infertile men group encompassed the inability to conceive for at least 24 months with the same female partner. For both infertile and fertile men, additional criteria included the absence of a medical history of conditions such as cancer, mumps orchitis, adenoma, cardiovascular diseases, nephritis, hepatitis, diabetes, eating disorders, inflammation in the genitourinary system, Klinefelter’s syndrome, cryptorchidism, testicular torsion, varicocele, sexually transmitted diseases, obesity, neurological disorders, tuberculosis, or any history of injury, damage, and/or surgery in the genitourinary system, scrotum, or groin area. Furthermore, all participants were required to have no current acute infectious diseases with high fever, no leukocytospermia, and/or presence of bacteria in semen. Additionally, for the fertile group, the key inclusion criterion was having at least one child under the age of three years. Information on potential confounding factors such as dietary habits, body mass index, smoking status etc. was not collected during recruitment. This limitation resulted from the retrospective and exploratory nature of the study design. As such, these variables were not included as formal inclusion or exclusion criteria, nor were they statistically controlled for in the analyses.

The ejaculates were collected via masturbation into sterile polyethylene containers after 3–5 days of sexual abstinence. After liquefaction (maximum 60 min at 37 °C), a standard semen analysis was conducted. It encompassed parameters such as semen volume, pH, and sperm viability. Computer-assisted semen analysis (SCA Motility and Concentration software version 6.5.0.5, Microptic SL, Barcelona, Spain) was used to assess additional quality parameters, including total sperm count, sperm concentration, total motility, progressive motility, and sperm morphology. Additionally, the concentration of morphologically abnormal sperm was calculated based on sperm concentration and the percentage of sperm with normal morphology. To obtain seminal plasma, after standard semen analysis, ejaculates were centrifuged for 10 min at room temperature at 3500 × g. Then, seminal plasma was aliquoted into smaller portions and stored at − 86 °C at the Wroclaw Medical University Biobank until the commencement of the study. All samples were processed and analyzed anonymously.

Based on the standard semen analysis, among infertile patients, the following groups have been distinguished following the WHO recommendations from 2021^1^: teratozoospermic (T, n = 73, < 4% of spermatozoa had normal morphology), asthenozoospermic (A, n = 12, < 32% of sperm demonstrated progressive motility), azoospermic (Azoo, n = 23, lack of spermatozoa in the semen), infertile normozoospermic (NI, n = 16, ejaculate parameter values are correct and meet WHO criteria for normozoospermia) and mixed groups: asthenoteratozoospermic (AT, n = 68, < 32% of sperm demonstrated progressive motility and < 4% of spermatozoa had normal morphology), oligoteratozoospermic (OT, n = 16, sperm count < 15 × 10^6^ mL^–1^ and < 4% of spermatozoa had normal morphology), oligoasthenoteratozoospermic (OAT, n = 42, sperm count < 15 × 10^6^ mL^–1^, < 32% of sperm demonstrated progressive motility and < 4% of spermatozoa had normal morphology) (Table 1). The control group (F, n = 22) consists of men with proven fertility (fertile healthy men who have at least one child in age lower than three years).

**Table 1:** Characteristic of semen quality parameters of the examined groups of infertile men.

Group	Parameter					
	Sperm concentration [× 10^6^/mL]	Sperm count per ejaculate [× 10^6^]	Sperm viability [%]	Sperm total motility [%]	Sperm progressive motility [%]	Sperm normal morphology [%]
	Me (Q1–Q3)					
T (n = 73)	50.99 (31.40–73.69)	216.61 (170.46–287.74)	65.00 (57.00–77.00)	64.59 (56.94–73.95)	48.71 (38.79–56.65)	1.44 (0.88–2.49)
A (n = 12)	47.24 (32.18–76.25)	156.92 (116.35–305.57)	73.00 (67.00–77.00)	55.69 (48.05–70.37)	18.51 (9.30–21.24)	4.57 (4.43–5.76)
Azoo (n = 23)	0.00 (0.00–0.00)					
NI (n = 16)	44.86 (28.68–74.19)	217.09 (159.04–358.50)	70.00 (49.00–77.00)	68.90 (50.97–74.99)	47.08 (37.32–60.05)	4.47 (4.05–6.39)
AT (n = 68)	37.96 (25.37–61.35)	151.22 (87.51–220.82)	43.50 (35.50–62.00)	40.82 (32.28–52.86)	19.58 (12.40–25.71)	0.99 (0.46–1.88)
OT (n = 16)	9.62 (7.24–11.72)	43.12 (31.88–77.13)	54.50 (49.50–62.00)	52.21 (46.85–61.34)	38.85 (34.49–51.19)	1.00 (0.96–2.41)
OAT (n = 42)	6.88 (4.49–12.30)	34.56 (21.33–56.62)	35.00 (30.00–43.00)	32.17 (26.99–39.64)	15.67 (11.28–21.37)	0.93 (0.44–1.49)

A: asthenozoospermic group, Azoo: azoospermic group, AT: asthenoteratozoospermic group, Me–median, NI: normozoospermic infertile group, OAT: oligoasthenoteratozoospermic group, OT: oligoteratozoospermic group, Q1-Q3: interquartile range, T–teratozoospermic group.

### Chemicals and reagents

Pentadecanoic acid (C15:0), n-hexane, ethanol, methanol, 2,6-Di-*tert*-butyl-4-methylphenol (BHT), acetyl chloride, and Supelco 37 Component FAME Mix were purchased from Merck (Darmstadt, Germany). A 1 mg/mL C15:0 solution and an 11 mg/mL BHT solution were prepared by dissolving 10 mg of C15:0 and 110 mg of BHT in 10 mL of n‑hexane, respectively.

### Seminal plasma PUFAs extraction and instrumental analysis

PUFAs, including linoleic acid, α-linolenic acid, γ-linolenic acid, arachidonic acid, eicosapentaenoic acid, and docosahexaenoic acid, were extracted from seminal plasma utilizing a modified procedure developed by Cabrini et al.^39^. The initial extraction involved mixing 75 μL of seminal plasma and 5 μL of the 1 mg/mL C15:0 solution with 350 μL of an n-hexane/ethanol mixture (5:2, v/v) through brief vortexing for approximately 1 min. The mixture was then centrifuged at 4000 rpm for 5 min. The upper organic phase was collected, and the separated lower aqueous phase was re-extracted three times with 250 μL of n-hexane. To the collected extracts, 10 μL of 11 mg/mL BHT was added to prevent oxidation. The extracts were evaporated under a nitrogen (N_2_) atmosphere at 30 °C for approximately 5 min.

The subsequent conversion of PUFAs to fatty acid methyl esters (FAMEs) was achieved following a developed protocol. The evaporated samples were treated with 1.5 mL of methanol and 200 μL of acetyl chloride, heated at 85 °C for 3 h with stirring every 30 min. After cooling, 500 μL of n-hexane and 500 μL of deionized water were added to the samples, which were then vortexed for approximately 1 min and centrifuged at 1000 rpm for 5 min. The upper layer was collected. The extraction procedure was repeated with an additional portion of n-hexane (500 μL), and the collected extracts were evaporated under a N_2_ atmosphere at 30 °C for approximately 5 min. Finally, dry extracts were resuspended in 100 μL of n-hexane and mixed for 1 min.

The instrumental analysis of FAMEs extracted from seminal plasma was conducted using a 7890B gas chromatograph (Agilent Technologies, Santa Clara, USA), equipped with a PAL combi-xt autosampler (CTC, Zwingen, Switzerland), and coupled to a 7000D triple quadrupole mass spectrometer (Agilent Technologies, Santa Clara, USA). Chromatographic separation was performed on a 30 m × 0.25 mm × 0.25 mm FastFAME capillary column (Agilent Technologies, Santa Clara, USA). Helium was used as the carrier gas at a flow rate of 1 mL/min. The chromatographic temperature program started at 50 °C for 2.5 min, followed by a ramp of 35 °C/min to 194 °C, and then 3.5 °C/min to a final temperature of 240 °C, which was held for 1 min. The ion source temperature was maintained at 220 °C, with an electron energy of 60 eV. The auxiliary heater was set to 240 °C. The inlet was operated in pulsed splitless mode with an initial temperature of 70 °C, held for 0.1 min, and ramped at 600 °C/min to 350 °C, where it was held for 5 min. The injection pulse pressure was 25 psi for 0.75 min, followed by a purge flow of 50 mL/min to the split vent at 0.76 min. The total run time was 26.56 min.

Analyses were performed in multiple reaction monitoring (MRM) mode to detect specific transitions for each FAME:

- **C18:2 n-6 *cis***: Quantifier 67 → 41, Qualifier 294 → 81
- **C18:3 n-3**: Quantifier 95 → 67, Qualifier 149 → 93.1
- **C18:3 n-6**: Quantifier 79 → 77, Qualifier 194 → 120.3
- **C20:4 n-6**: Quantifier 79 → 77, Qualifier 80 → 51
- **C20:5 n-3**: Quantifier 79 → 77, Qualifier 93 → 77
- **C22:6 n-3**: Quantifier 79 → 77, Qualifier 119 → 91

Peak identification and quantification of PUFA concentrations were performed using external standards from the Supelco 37 Component FAME Mix. The concentration of individual PUFA in the prepared sample was determined based on a five-point calibration curve (standard dilutions in n-hexane at ratios of 1:100, 1:200, 1:300, 1:400, and 1:500). The obtained values were then normalized to PUFA concentrations per milliliter of seminal plasma (μg/mL). PUFA ratios were calculated by dividing individual PUFA concentrations in seminal plasma.

To determine the optimal volume of seminal plasma required for the detection of all selected PUFAs, a series of preliminary experiments was conducted. PUFAs extraction was performed using 50, 75, 100, and 150 µL of seminal plasma. The optimal volume, ensuring sufficiently distinct peaks for the identification of individual PUFAs, was found to be 75 µL. Method validation and quality control procedures were conducted to ensure the reliability of the results, including matrix effect analysis on quality control (QC) samples (mixed aliquots of each sample) determined every 10 samples during the whole sequence. PUFAs extraction and analysis were performed in two independent replicates. The extraction procedure was carried out by one person to ensure consistency and minimize variability in the process.

**Fig. 1:**
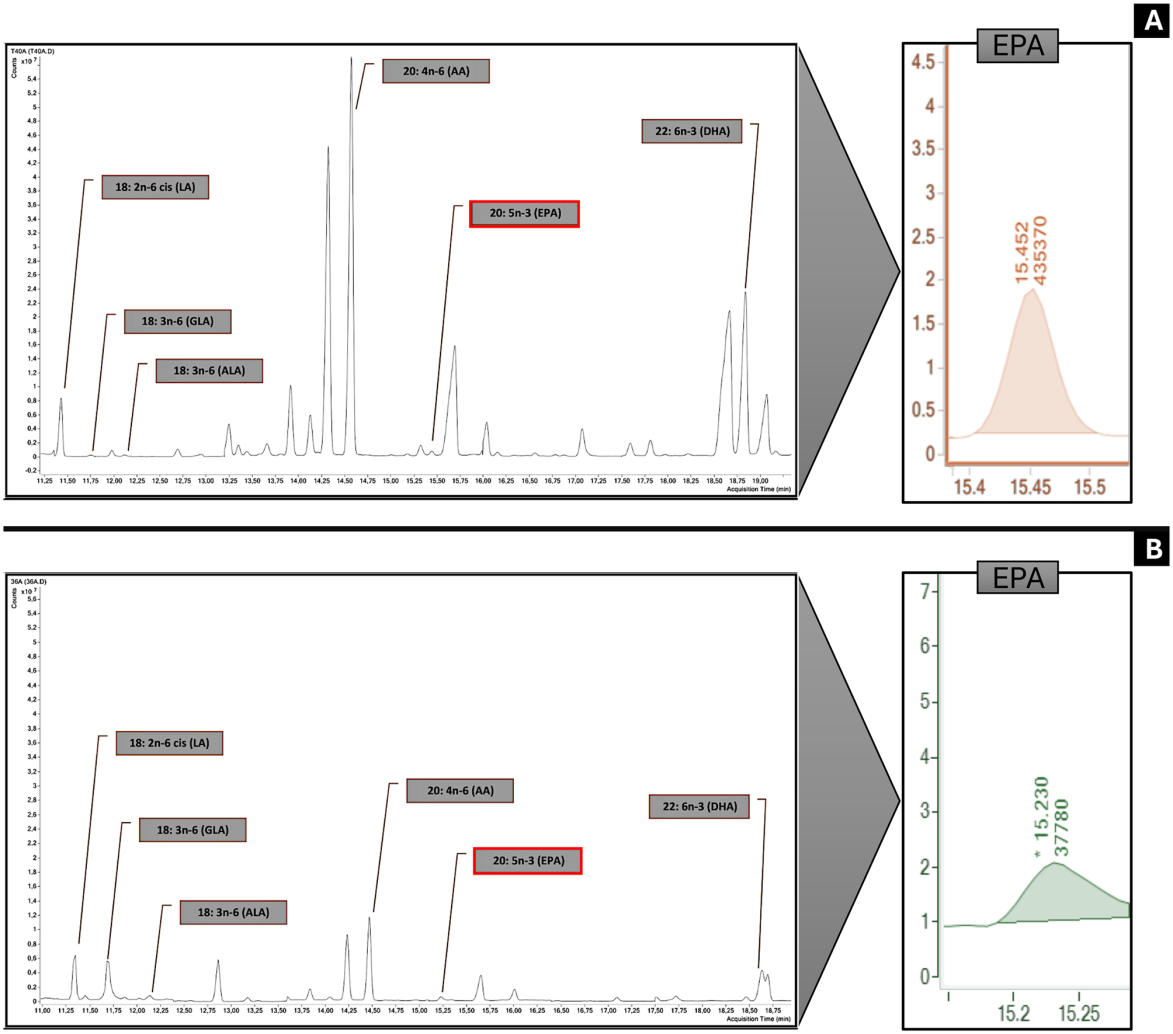
An example of two GC-chromatograms of seminal plasma PUFA composition: (**A**) infertile men; (**B**) fertile men. AA: arachidonic acid, ALA: α-linolenic acid, DHA: docosahexaenoic acid, EPA: eicosapentaenoic acid, GLA: γ-linolenic acid, LA: linoleic acid.

Two examples of GC-chromatograms showing seminal plasma PUFA composition of fertile and infertile men are shown in Fig. 1.

### Statistical analysis

The data were analyzed using Statistica 13.3 PL software (StatSoft Poland Sp. z o.o., Krakow, Poland). All results are presented as the median (Me) with the interquartile range (Q1–Q3). Data were assessed for normality using the Shapiro–Wilk test. Due to the non-normal distribution, nonparametric tests were employed for subsequent analyses. The concentrations of individual PUFAs in seminal plasma, along with their ratios, were compared between fertile and infertile men using the Mann–Whitney U test. The clinical relevance of the determined parameters was evaluated via receiver operating characteristic (ROC) curve analysis. Subsequently, differences in the concentrations of PUFAs and their ratios in seminal plasma among the infertile patient groups were examined using ANOVA rank test followed by Tukey’s post hoc test. Correlations between the examined parameters and standard semen parameters were assessed using Spearman’s rank test. The strength of the Spearman’s correlations was classified as follows: 0.0 ≤|R|≤ 0.2—no correlation; 0.2 <|R|≤ 0.4—weak correlation; 0.4 <|R|≤ 0.7—moderate correlation; 0.7 <|R|≤ 0.9—strong correlation; and 0.9 <|R|≤ 1.0—very strong correlation. A two-tailed *p*-value of less than 0.05 was considered significant. Box plots and heat maps were generated using GraphPad Prism (San Diego, USA).

The main text of the article presents statistically significant results, while findings that did not reach statistical significance or that had weak correlations are included in the Supplementary Materials (Tables S1–S5).

## Results

### Comparison of seminal plasma PUFA concentrations/ratios between infertile patients and fertile men

**Fig. 2:**
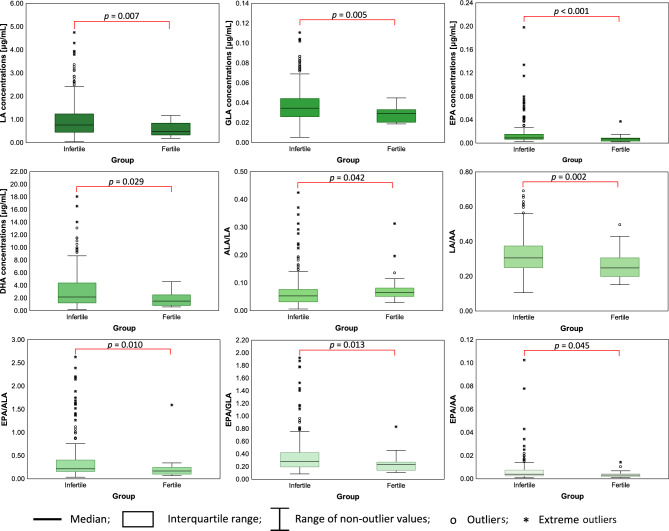
Comparison of PUFA concentrations and ratios between fertile and infertile men using box plots. A two-tailed *p*-value of less than 0.05 was considered significant: *p*-values from the Mann–Whitney U test are provided. DHA: docosahexaenoic acid, EPA: eicosapentaenoic acid, GLA: γ-linolenic acid, LA: linoleic acid.

Table 2 and Fig. 2 present significant differences (*p* < 0.05) in the seminal plasma concentrations of PUFAs and PUFA ratios between fertile and infertile men. Infertile men exhibited higher concentrations of PUFAs than those observed in fertile men; however, as shown by box plots (Fig. 2), significant differences were found only for LA, GLA, EPA, and DHA. Regarding PUFA ratios, ALA/LA was lower in the infertile group, whereas LA/AA, EPA/ALA, EPA/GLA, and EPA/AA were significantly higher when compared with those of the fertile control group. For the remaining parameters, the differences were not significant (*p* > 0.05), and their values are presented in Table S1 (Supplementary Materials).

**Table 2:** Comparison of the PUFA concentrations/ratios in seminal plasma between fertile and infertile men.

PUFA	PUFA concentration [μg/mL] Me (Q1-Q3)		*p*-value
	Fertile (n = 22)	Infertile (n = 250)	
LA (18:2 n-6 *cis*)	0.493 (0.335–0.838)	0.762 (0.454–1.238)	*p* = 0.007
GLA (18:3 n-6)	0.029 (0.021–0.033)	0.035 (0.026–0.044)	*p* = 0.005
EPA (20:5 n-3)	0.007 (0.004–0.008)	0.009 (0.007–0.015)	*p* < 0.001
DHA (22:6 n-3)	1.519 (0.083–2.508)	2.174 (1.238–4.390)	*p* = 0.029
ALA/LA	0.066 (0.051–0.081)	0.053 (0.032–0.076)	*p* = 0.042
LA/AA	0.248 (0.199–0.306)	0.305 (0.250–0.374)	*p* = 0.002
EPA/ALA	0.168 (0.100–0.246)	0.216 (0.158–0.404)	*p* = 0.010
EPA/GLA	0.230 (0.142–0.272)	0.283 (0.195–0.421)	*p* = 0.013
EPA/AA	0.003 (0.002–0.004)	0.004 (0.003–0.008)	*p* = 0.045

The Mann–Whitney U test was used to assess the differences between groups. A two-tailed *p*-value of less than 0.05 was considered significant. AA: arachidonic acid, ALA: α-linolenic acid, DHA: docosahexaenoic acid, EPA: eicosapentaenoic acid, GLA: γ-linolenic acid, LA: linoleic acid, Me: median, PUFA–polyunsaturated fatty acid, Q1–Q3: interquartile range.

### The results of ROC curves analysis

**Fig. 3:**
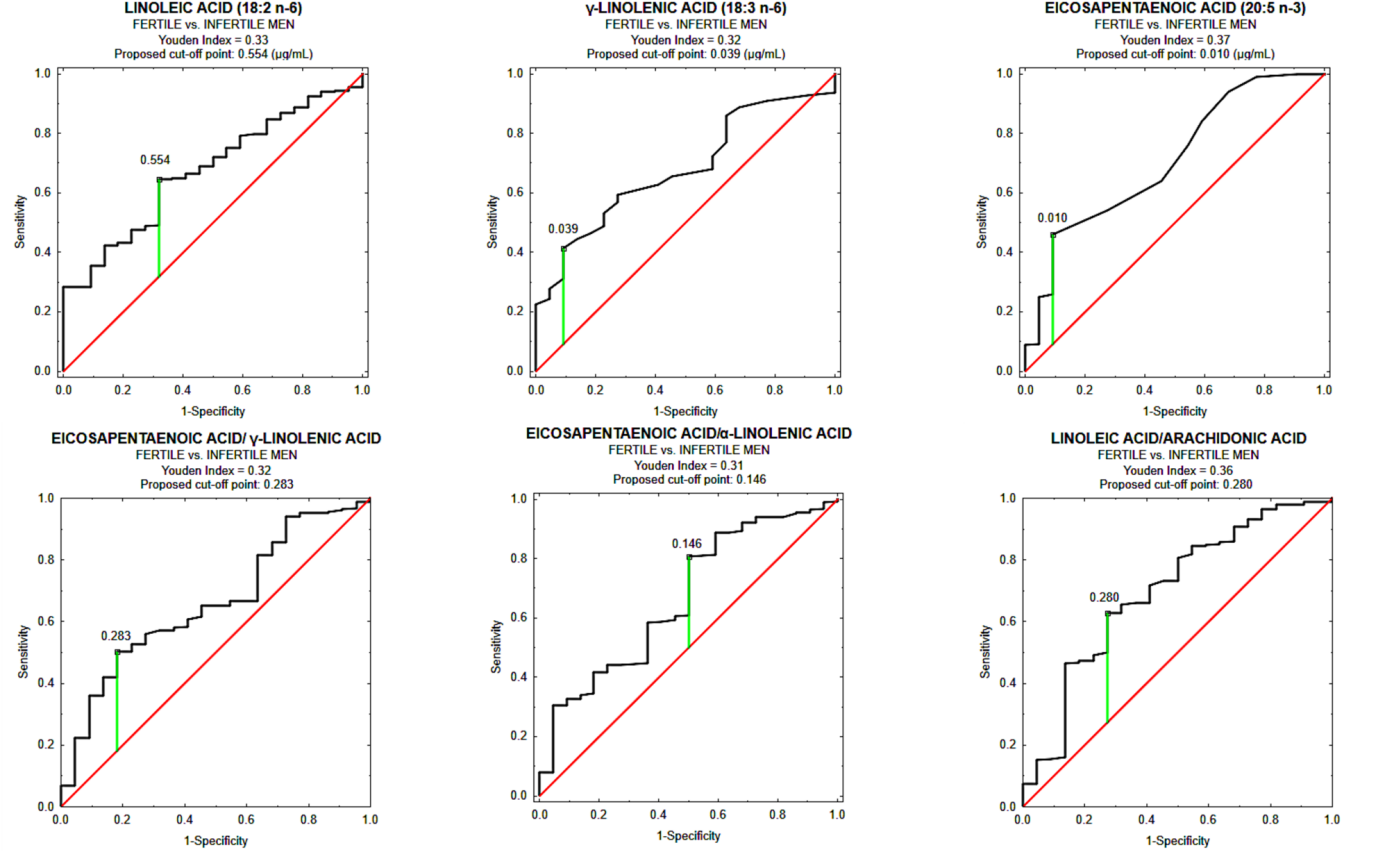
ROC curves for seminal plasma PUFA concentrations/ratios as potential biomarkers of male infertility.

To further assess the discriminatory potential of the analyzed PUFAs concentrations/ratios, ROC curve analyses were performed for parameters which values meet the criteria of statistical significance (p < 0.05) for examined groups differentiation and have acceptable diagnostic accuracy (AUC > 0.65), as presented in Fig. 3 and Table 3. Among these, LA, GLA, LA/AA, EPA/ALA, and EPA/GLA demonstrated limited diagnostic value, while EPA exhibited moderate discriminatory power. These results suggest that although individual markers may offer some level for differentiation between fertile and infertile men, their clinical utility remains limited without further validation, particularly considering the relatively small size of the fertile control group.

**Table 3:** Summary of the results of ROC curves analysis.

	AUC	AUC: 95% Confidence Interval	Cut-off point	Sensitivity [%]	Specificity [%]	*p*-value
LA (18:2 n-6 *cis*)	0.674	0.575–0.773	0.554	64.4	68.2	< 0.001
GLA (18:3 n-6)	0.679	0.583–0.776	0.039	41.2	90.9	< 0.001
EPA (20:5 n-3)	0.709	0.597–0.821	0.010	46.0	90.9	< 0.001
LA/AA	0.697	0.576–0.817	0.280	62.8	72.7	0.001
EPA/ALA	0.665	0.546–0.784	0.146	80.8	50.0	0.006
EPA/GLA	0.659	0.549–0.770	0.283	50.4	81.8	0.004

Data are given as AUC with 95% confidence interval. A *p*-value of less than 0.05 was considered significant. AA: arachidonic acid, ALA: α-linolenic acid, EPA: eicosapentaenoic acid, GLA: γ-linolenic acid, LA: linoleic acid.

The results of the ROC curves analysis for parameters with AUC values exceeding 0.65 and *p*-values below 0.05 are presented in Table 3. The results for the remaining parameters (AUC < 0.65) are provided in Supplementary Materials (Table S2).

### Comparison of seminal plasma PUFA concentrations/ratios between all examined groups of men

In PUFA concentrations, significant differences (*p* < 0.05) between examined groups were observed. LA concentrations were significantly higher in the T compared to the Azoo and F groups. GLA concentrations were significantly elevated in the T compared to the F group, while AA concentrations were higher in the T than in the Azoo group. Notably, EPA concentrations were significantly higher in the T compared to the Azoo, AT, and F groups. DHA concentrations were also markedly elevated in the T group relative to the Azoo, NI, AT, OT, OAT, and F groups.

Moreover, several PUFA ratios differed significantly between the studied groups. The T group exhibited a lower ALA/LA ratio than the A group, whereas LA/DHA, ALA/DHA, and GLA/DHA ratios were significantly lower compared to both the Azoo and OAT groups. Additionally, the ALA/GLA ratio was lower than in the AT group, while the AA/DHA ratio was significantly reduced relative to the Azoo, OT, OAT, and F groups. In the A group, the LA/DHA, GLA/DHA, and AA/DHA ratios were lower compared to the Azoo group. Moreover, Azoo patients exhibited a higher LA/DHA ratio compared to the A, NI, and F groups. The ALA/DHA ratio was elevated in the Azoo group relative to the AT and OT groups, whereas the GLA/DHA ratio was higher in the Azoo patients than in the NI, AT, OT, OAT, and F groups. Similarly, the AA/DHA ratio was higher in the Azoo group compared to the NI, AT, OAT, and F groups. Additionally, within the AT group, the AA/DHA and EPA/DHA ratios were significantly lower compared to the OAT group. These findings highlight the presence of specific lipidomic profiles associated with distinct forms of male infertility.

**Fig. 4:**
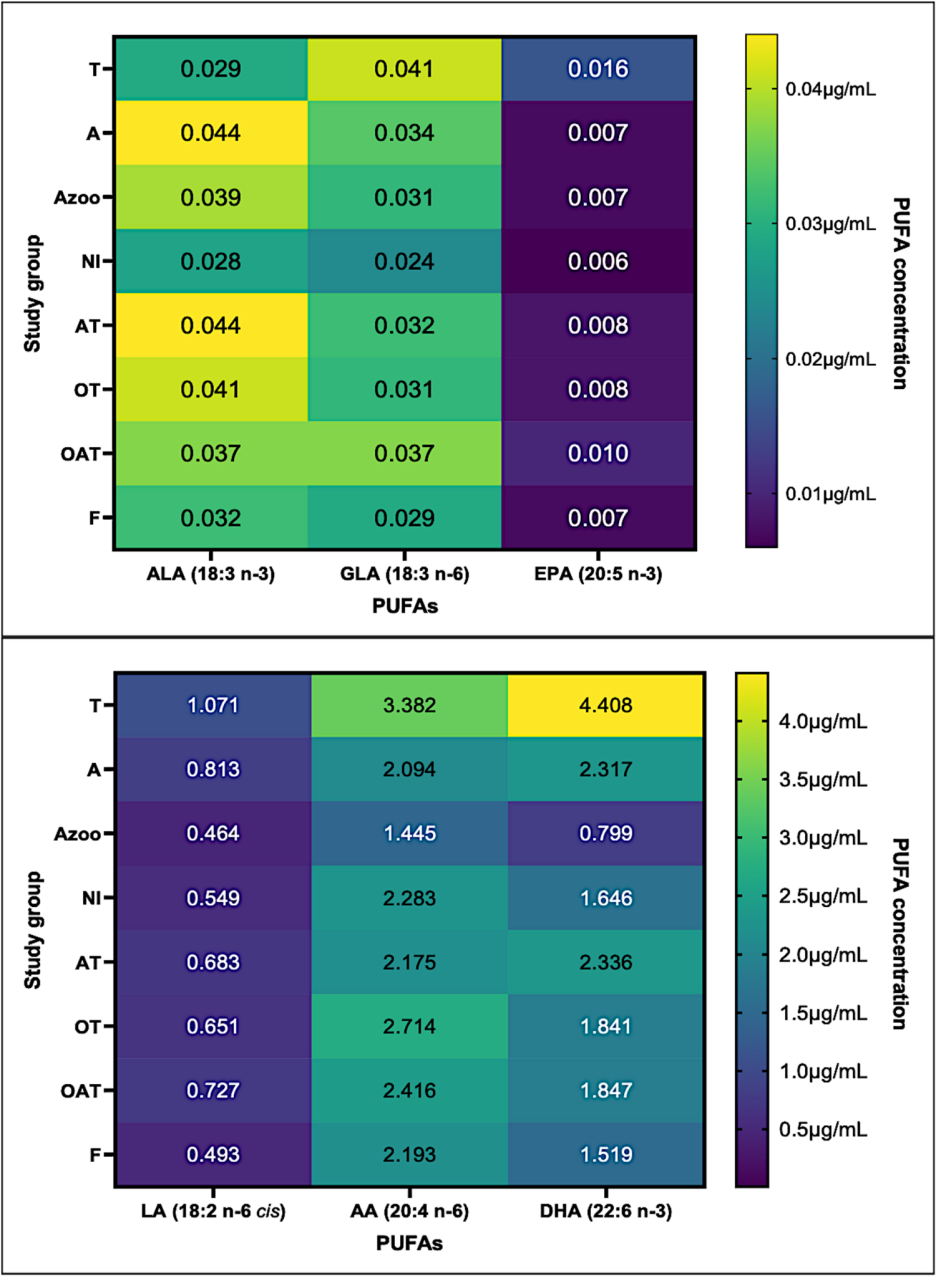
Comparison of PUFA concentrations between fertile and infertile men using a heat map. T vs. Azoo, p = 0.008; T vs. F, p = 0.027; T vs. F, p = 0.042; T vs. Azoo, p = 0.016; T vs Azoo, p = 0.017; T vs. AT, p < 0.001; T vs. F, p = 0.026; T vs. Azoo, p < 0.001; T vs. NI, p = 0.034; T vs. AT, p < 0.001; T vs. OT, p = 0.032; T vs. OAT, p < 0.001; T vs. F, p < 0.001. A: asthenozoospermic group, AA: arachidonic acid, ALA: α-linolenic acid, Azoo: azoospermic group, AT: asthenoteratozoospermic group, DHA: docosahexaenoic acid, EPA: eicosapentaenoic acid, F: fertile group, GLA: γ-linolenic acid, LA: linoleic acid, Me–median, NI: normozoospermic infertile group, OAT: oligoasthenoteratozoospermic group, OT: oligoteratozoospermic group, PUFA(s) : polyunsaturated fatty acid(s), T–teratozoospermic group. Median values are given in the boxes.

The median concentrations of PUFAs and PUFA ratios in seminal plasma, for which at least one significant difference was detected between the studied groups of men, are presented in Tables 4, 5 and Fig. 4. Due to the considerable variation in concentration magnitudes among individual PUFAs, a single heat map did not adequately reflect the differences between groups. Thus, two separate heat maps were created, gathering the parameters with similar PUFA concentration/ratio values, to more effectively highlight and compare the variations between studied groups. Parameters for which the obtained result did not differ significantly between examined groups (*p* > 0.05) are presented in Supplementary Materials (Table S3).

**Table 4:** Comparison of the concentrations of PUFAs in seminal plasma between the examined groups of men.

Group	PUFA					
	LA (18:2 n-6 *cis*) [μg/mL]	ALA (18:3 n-3) [μg/mL]	GLA (18:3 n-6) [μg/mL]	AA (20:4 n-6) [μg/mL]	EPA (20:5 n-3) [μg/mL]	DHA (22:6 n-3) [μg/mL]
	Me (Q1–Q3)					
T	**1.071**^**a,b**^ **(0.724–1.750)**	0.029 (0.019–0.065)	**0.041**^**c**^ **(0.032–0.051)**	**3.382**^**d**^ **(2.061–5.653)**	**0.016**^**e,f,g**^ **(0.009–0.027)**	**4.408**^** h**^^**,i,j,k,l,m**^ **(2.528–5.709)**
A	0.813 (0.487–1.173)	0.044 (0.036–0.055)	0.034 (0.031–0.040)	2.094 (1.593–3.987)	0.007 (0.006–0.009)	2.317 (1.503–3.532)
Azoo	0.464 (0.327–0.639)	0.039 (0.030–0.044)	0.031 (0.023–0.040)	1.445 (0.972–2.408)	0.007 (0.006–0.008)	0.799 (0.502–1.093)
NI	0.549 (0.393–1.125)	0.028 (0.019–0.043)	0.024 (0.021–0.036)	2.283 (1.429–3.291)	0.006 (0.005–0.009)	1.646 (1.234–3.586)
AT	0.683 (0.443–1.189)	0.044 (0.030–0.065)	0.032 (0.025–0.040)	2.175 (1.297–4.408)	0.008 (0.006–0.010)	2.336 (1.252–4.325)
OT	0.651 (0.467–1.750)	0.041 (0.028–0.049)	0.031 (0.025–0.042)	2.714 (1.538–5.329)	0.008 (0.005–0.016)	1.841 (1.301–3.016)
OAT	0.727 (0.354–1.080)	0.037 (0.026–0.045)	0.037 (0.026–0.045)	2.416 (1.557–3.505)	0.010 (0.008–0.020)	1.847 (1.147–2.320)
F	0.493 (0.335–0.838)	0.032 (0.023–0.048)	0.029 (0.021–0.033)	2.193 (1.480–3.155)	0.007 (0.004–0.008)	1.519 (0.083–2.508)

^a^T vs. Azoo, *p* = 0.008.

^b^T vs. F, *p* = 0.027.

^c^T vs. F, *p* = 0.042.

^d^T vs. Azoo, *p* = 0.016.

^e^T vs Azoo, *p* = 0.017.

^f^T vs. AT, *p* < 0.001.

^g^T vs. F, *p* = 0.026.

^h^T vs. Azoo, *p* < 0.001.

^i^T vs. NI, *p* = 0.034.

^j^T vs. AT, *p* < 0.001.

^k^T vs. OT, *p* = 0.032.

^l^T vs. OAT, *p* < 0.001.

^m^T vs. F, *p* < 0.001.

The ANOVA followed by Tukey’s test was used to assess the differences between groups. A two-tailed *p*-value of less than 0.05 was considered significant.

A: asthenozoospermic group, AA: arachidonic acid, ALA: α-linolenic acid, Azoo: azoospermic group, AT: asthenoteratozoospermic group, DHA: docosahexaenoic acid, EPA: eicosapentaenoic acid, F: fertile group, GLA: γ-linolenic acid, LA: linoleic acid, Me–median, NI: normozoospermic infertile group, OAT: oligoasthenoteratozoospermic group, OT: oligoteratozoospermic group, PUFA: polyunsaturated fatty acid, Q1–Q3: interquartile range, T–teratozoospermic group. Values with statistical significance are indicated in bold font.

**Table 5:** Comparison of the PUFAs ratios in seminal plasma between the examined groups of men.

Ratio	Group							
	T	A	Azoo	NI	AT	OT	OAT	F
	Me (Q1–Q3)							
ALA/LA	**0.033 **^**a**^ **(0.022–0.058)**	0.061 (0.040–0.096)	0.072 (0.063–0.093)	0.041 (0.028–0.089)	0.061 (0.039–0.092)	0.046 (0.040–0.067)	0.052 (0.036–0.074)	0.066 (0.051–0.081)
LA/DHA	**0.281 **^**b,c**^ **(0.208–0.357)**	**0.315 **^**d**^ **(0.284–0.407)**	**0.533 **^**e,f,g**^ **(0.470–0.651)**	0.360 (0.297–0.407)	0.331 (0.253–0.373)	0.428 (0.339–0.549)	0.401 (0.324–0.569)	0.305 (0.233–0.458)
ALA/GLA	**0.872**^h^ **(0.536–1.375)**	1.376 (0.974–1.717)	1.198 (0.872–1.486)	0.998 (0.835–1.603)	1.370 (0.980–1.750)	1.152 (0.943–1.516)	1.197 (0.793–1.502)	1.174 (0.935–1.620)
EPA/ALA	**0.449 **^**i,j,k,l**^ **(0.245–0.880)**	0.158 (0.118–0.183)	0.185 (0.151–0.275)	0.202 (0.144–0.251)	0.188 (0.131–0.254)	0.207 (0.158–0.292)	0.265 (0.156–0.444)	0.168 (0.100–0.246)
ALA/DHA	**0.009**^m^^**,n**^ **(0.005–0.015)**	0.024 (0.012–0.033)	**0.039 **^**o,p**^ **(0.030–0.078)**	0.015 (0.008–0.029)	0.019 (0.013–0.032)	0.022 (0.015–0.026)	0.020 (0.014–0.036)	0.023 (0.013–0.036)
EPA/GLA	**0.385 **^**r**^ **(0.255–0.708)**	0.201 (0.148–0.298)	0.242 (0.166–0.295)	0.265 (0.156–0.326)	0.242 (0.178–0.336)	0.291 (0.174–0.404)	0.281 (0.216–0.563)	0.230 (0.142–0.272)
GLA/DHA	**0.010**^s^^**,t**^ **(0.007–0.016)**	**0.017 **^**u**^ **(0.009–0.022)**	**0.040 **^**w,x,y,z,aa**^ **(0.020–0.056)**	0.016 (0.011–0.021)	0.016 (0.009–0.024)	0.018 (0.015–0.022)	0.021 (0.014–0.032)	0.018 (0.012–0.027)
AA/DHA	**0.892 **^**ab,ac,ad,ae**^ **(0.683–1.098)**	**0.917 **^**af**^ **(0.679–1.455)**	**1.872 **^**ag,ah,ai,aj**^ **(1.584–2.355)**	1.142 (0.900–1.503)	**1.067 **^**ak**^ **(0.892–1.264)**	1.403 (1.110–1.803)	1.419 (1.119–1.679)	1.330 (0.944–1.636)
EPA/DHA	0.005 (0.002–0.008)	0.003 (0.002–0.005)	0.008 (0.006–0.012)	0.004 (0.002–0.006)	**0.004 **^**al**^ **(0.002–0.006)**	0.005 (0.003–0.008)	0.007 (0.004–0.013)	0.004 (0.002–0.007)

^a^T vs. AT, *p* = 0.001.

^b^T vs. Azoo, *p* < 0.001.

^c^T vs. OAT, *p* < 0.001.

^d^A vs. Azoo, *p* = 0.024.

^e^Azoo vs. NI, *p* = 0.030.

^f^Azoo vs. AT, *p* < 0.001.

^g^Azoo vs. F, *p* = 0.001.

^h^T vs. AT, *p* = 0.011.

^i^T vs. Azoo, *p* = 0.017.

^j^T vs. AT, *p* < 0.001.

^k^T vs. OAT, *p* = 0.035.

^l^T vs. F, *p* = 0.034.

^m^T vs. Azoo, *p* < 0.001.

^n^T vs. OAT, *p* < 0.001.

^o^Azoo vs. AT, *p* = 0.032.

^p^Azoo vs. OT, *p* = 0.040.

^r^T vs. AT, *p* < 0.001.

^s^T vs. Azoo, *p* < 0.001.

^t^T vs. OAT, *p* < 0.001.

^u^Azoo vs. A, *p* < 0.001.

^w^Azoo vs. NI, *p* < 0.001.

^x^Azoo vs. AT, *p* < 0.001.

^y^Azoo vs. OT, *p* < 0.001.

^z^Azoo vs. OAT, *p* < 0.001.

^aa^Azoo vs. F, *p* < 0.001.

^ab^T vs. Azoo, *p* < 0.001.

^ac^T vs. OT, *p* = 0.004.

^ad^T vs. OAT, *p* < 0.001.

^ae^T vs. F, *p* = 0.005.

^af^A vs. Azoo, *p* < 0.001.

^ag^Azoo vs. NI, *p* < 0.001.

^ah^Azoo vs. AT, *p* < 0.001.

^ai^Azoo vs. OAT, *p* = 0.009.

^aj^Azoo vs. F, *p* = 0.001.

^ak^AT vs. OAT, *p* = 0.006.

^al^AT vs. OAT, *p* = 0.009.

The ANOVA followed by Tukey’s test was used to assess the differences between groups. A two-tailed *p*-value of less than 0.05 was considered significant. A: asthenozoospermic, AA: arachidonic acid, ALA: α-linolenic acid, Azoo: azoospermic group, AT: asthenoteratozoospermic group, DHA: docosahexaenoic acid, EPA: eicosapentaenoic acid, F: fertile group, GLA: γ-linolenic acid, LA: linoleic acid, Me: median, NI: normozoospermic infertile group, OAT: oligoasthenoteratozoospermic group, OT: oligoteratozoospermic group, PUFA: polyunsaturated fatty acid, Q1–Q3: interquartile range, T: teratozoospermic group. Values with statistical significance are indicated in bold font.

### Correlations between seminal plasma PUFA concentrations/ratios and standard semen parameters

Correlation coefficients for PUFA concentrations and ratios exhibiting at least moderate correlations (|R|> 0.4, *p* < 0.05) with one or more standard semen parameters are presented in Table 6, while the remaining correlations are provided in Supplementary Materials (Table S4).

**Table 6:** Correlations between PUFA concentrations/ratios and standard semen parameters.

PUFA	Parameter													
	Sperm concentration [× 10^6^/mL]		Sperm count per ejaculate [× 10^6^]		Sperm viability [%]		Sperm total motility [%]		Sperm progressive motility [%]		Sperm normal morphology [%]		Concentration of morphologically abnormal sperm [× 10^6^/mL]	
	R	*p*	R	*p*	R	*p*	R	*p*	R	*p*	R	*p*	R	*p*
DHA (22:6 n-3)	**0.46**	** < 0.001**	**0.41**	** < 0.001**	0.30	< 0.001	0.38	< 0.001	0.38	< 0.001	0.14	0.024	**0.46**	** < 0.001**
LA/DHA	− **0.52**	** < 0.001**	− **0.44**	** < 0.001**	− 0.39	< 0.001	− 0.38	< 0.001	− 0.30	< 0.001	− 0.11	0.080	− **0.52**	** < 0.001**
ALA/DHA	− **0.42**	** < 0.001**	− 0.36	< 0.001	− 0.28	< 0.001	− 0.38	< 0.001	− **0.43**	** < 0.001**	− 0.13	0.034	− **0.42**	** < 0.001**
GLA/DHA	− **0.50**	** < 0.001**	− **0.41**	** < 0.001**	− 0.30	< 0.001	− 0.36	< 0.001	− 0.35	< 0.001	− 0.10	0.107	− **0.50**	** < 0.001**
AA/DHA	− **0.60**	** < 0.001**	− **0.55**	** < 0.001**	− 0.35	< 0.001	− 0.40	< 0.001	− 0.35	< 0.001	− 0.18	0.005	− **0.61**	** < 0.001**
EPA/DHA	− **0.42**	** < 0.001**	− 0.29	< 0.001	− 0.24	< 0.001	− 0.21	< 0.001	− 0.14	0.022	− 0.15	0.017	− **0.42**	** < 0.001**

Spearman’s rank test was used to assess the correlations between analyzed parameters, and a *p*-value of less than 0.05 was considered significant. Moderate, and higher, (0.4 <|R|, p < 0.05) significant correlations are marked in bold. AA: arachidonic acid, ALA: α-linolenic acid, DHA: docosahexaenoic acid, EPA: eicosapentaenoic acid, GLA: γ-linolenic acid, LA: linoleic acid, PUFA: polyunsaturated fatty acid.

Correlation analysis revealed that among individual PUFAs, DHA exhibited moderate positive correlations with key semen quality parameters, including sperm concentration, total sperm count per ejaculate, and the concentration of morphologically abnormal sperm. In contrast, PUFA ratios generally showed predominantly negative correlations with semen quality parameters. Specifically, the LA/DHA, GLA/DHA, and AA/DHA ratios exhibited moderate negative correlations with sperm concentrations, sperm count per ejaculate and concentrations of morphologically abnormal sperm. Additionally, the ALA/DHA and EPA/DHA ratios demonstrated moderate negative correlations with sperm concentrations and concentrations of morphologically abnormal sperm. The ALA/DHA ratio was also moderately negatively correlated with sperm progressive motility. Other parameters exhibited weak or no correlations.

## Discussion

Male reproductive potential is not solely determined by semen parameters such as sperm concentration, progressive motility, and morphology, as infertility can also be observed in men with normozoospermia^12,13,40^. Thus, there is an urgent need to identify novel biomarkers for male infertility, to uncover and understand the potential mechanisms leading to a decline in male reproductive capacity and to optimize existing and/or develop and implement new analytical methods. Seminal plasma is a source of many biomarkers associated with male reproductive potential, and their examination provides variety of new information about the mechanisms leading to reduced semen quality and, consequently, reduced male fertility. In the light of increasing interest in the role of PUFAs present in ejaculate in the context of male infertility causes, driven by a deeper exploration of the role of environmental factors and diet in maintaining semen quality, limited diagnostic capabilities, and evolving diagnostic criteria for male infertility, the present research fits into this trend. However, it is important to emphasize that the present study represents an exploratory contribution to research aimed at identifying male infertility biomarkers and delineates future directions for investigations involving larger cohorts of men. Nevertheless, the use of presented methodological approach provides a strong foundation for further studies with potential clinical applicability. The study was carried out thanks to an optimization of a method for the extraction and analysis of seminal plasma PUFA concentrations using validated GC–MS/MS method, which made it possible to determine and subsequently compare the concentrations of selected PUFAs (LA, ALA, GLA, AA, EPA, DHA) and their ratios in seminal plasmas from fertile and infertile men (both as a whole and stratified into subgroups according to the 2021 WHO criteria (1)), and to assess their potential clinical utility as biomarkers of male infertility. Additionally, the study examined correlations between selected seminal plasma parameters and standard semen parameters (sperm concentration, total sperm count, viability, motility, progressive motility, and morphology) and analyzed the impact of introducing new diagnostic criteria for male infertility by comparing outcomes in men groups classified according to the WHO standards from 2010 and 2021.

Although GC has been widely used in the metabolic profiling of various biological materials, its application to human seminal plasma analysis is relatively rare and requires substantial sample volumes and extended processing times, thereby limiting its practical applicability in clinical diagnostics^41–46^. To the best of our knowledge, the present study is the first to employ GC–MS/MS for the analysis of PUFA concentrations in human seminal plasma, whereas previous methods have focused on the use of gas chromatography-flame ionization detection (GC-FID), gas chromatography-mass spectrometry (GC–MS), and liquid chromatography^24,41,46–56^. GC–MS/MS provides the highest precision and selectivity and, through the use of two stages of mass analysis, allows for the detection of molecules at very low concentrations, suggesting it may be an ideal tool for studying biomarkers and mechanisms of diseases, including infertility^17,57,58^. Additionally, previous studies on the analysis of PUFAs in human seminal plasma have relied on various extraction methods, often requiring relatively large sample volumes (up to 300 μL; on average, 150 µL), with some protocols involving up to 16 h of incubation^24,41,43,46,52–54,56^. Here GC–MS/MS methodology was developed and optimized, which enabled faster analysis while requiring significantly smaller sample volumes. In our protocol (based on the n-hexane/ethanol method by Cabrini et al.^39^), we used only 75 μL of seminal plasma, with an incubation time during PUFAs esterification of 3 h, which is of particular importance in potential routine laboratory tests, where the available sample volume is often limited and the time required for analysis is critical. The optimization of the extraction method was conducted through a series of preliminary experiments, which involved using different volumes of seminal plasma (50, 75, 100, and 150 μL) from men with varying semen parameters, aimed at ensuring detectability in each patient group and the use of GC–MS/MS, which allowed for the detection of substances at very low concentrations in the sample.

In contrast to the extensive body of research on the PUFA composition of spermatozoa membranes, their analysis in human seminal plasma remains a relatively underexplored field, despite evidence indicating that these fatty acids are present in substantial quantities in both spermatozoa membranes and seminal plasma and that alterations in their content have been associated with changes in semen quality and fertilization potential^21^. Moreover, the mechanisms underlying these variations, as well as their impact on male fertility, have not yet been fully elucidated^21^. The exploratory comparative analysis of PUFA concentrations and ratios in seminal plasma between fertile and infertile men conducted in the present study revealed significant differences in 9 out of 21 examined parameters. The concentrations of LA, GLA, EPA, and DHA were significantly higher in the infertile group, as were the ratios of LA/AA, EPA/ALA, EPA/GLA, and EPA/AA, whereas the ALA/LA ratio was lower in this group. Additionally, differences were observed for most parameters between the groups of men classified according to the 2021 WHO criteria^1^. It is suggested that alterations in PUFA concentrations in seminal plasma may result from their release from sperm cell membranes due to the factors such as oxidative stress, which may lead to spermatozoa membrane damage^21^. Our findings support this hypothesis, especially because the teratozoospermic group, characterized by abnormal sperm morphology, predominantly exhibits higher PUFA concentrations compared to the other groups. Moreover, in the azoospermic group, in which spermatozoa are entirely absent in the ejaculate, seminal plasma was generally characterized by lower PUFA levels. In turn, spermatozoa membrane damage and the increased release of PUFAs may be associated with various pathological conditions and/or environmental factors that may induce oxidative stress and/or inflammatory processes^21^. However, the observed differences might also have other underlying causes. It is also possible that the persistence of elevated PUFA concentrations in seminal plasma may result from impaired spermatogenesis and defective sperm maturation, leading to the release of PUFAs unused in this processes into this body fluid^28^. Moreover, the spermatozoa in the testis normally exhibit high PUFA levels, which decrease during the functional remodeling of their membranes during epididymal transit. Therefore, if this process is disrupted, it may result in altered PUFA contents in both cell membranes and seminal plasma^49^. Both LA (omega-6) and ALA (omega-3) are delivered to the human organism with food, serving as precursors for other PUFAs^21^. Notably, harmful health effects associated with diminished omega-3/omega-6 ratio in the diet, including male infertility, have been documented in literature^59^. Moreover, some authors demonstrated that omega-3 PUFAs supplementation, particularly DHA, may positively influence semen quality^21,42,48^. Also, it has been demonstrated that the omega-6 to omega-3 ratio in spermatozoa membranes is higher in infertile compared to fertile men^21^. Therefore, it is possible that the reduced ALA/LA ratio and increased LA concentration in seminal plasma of infertile men directly reflect their content in spermatozoa membranes and/or are associated with reduced intake of ALA and/or increased consumption of LA in the diet. It is also suggested that PUFA levels in the blood may reflect their concentrations in seminal plasma^55^. The increased LA/AA ratio in the infertile men group is most likely directly attributed to the observed higher concentration of LA in seminal plasma of infertile patients, while no significant differences in AA levels were found between the fertile and infertile men. However, it is important to consider that AA is enzymatically converted into pro-inflammatory eicosanoids, which can induce inflammation within the male reproductive organs, thereby affecting spermatozoa function^21^. This could explain the observed lack of significant differences in AA concentration in seminal plasma between fertile and infertile men and the association between the higher LA/AA ratio and male infertility. It should also be noted that both the conversion of omega-3 to anti-inflammatory metabolites and omega-6 to pro-inflammatory metabolites involve the same enzymes^21^. Therefore, the increased EPA/ALA, EPA/AA, EPA/GLA ratios and EPA concentration in seminal plasma of infertile men may suggest that the enzymatic conversion of EPA into anti-inflammatory metabolites is impaired. It is also possible that the enzymes responsible for this conversion are diverted to produce pro-inflammatory metabolites from the excess available omega-6 PUFAs, which affect spermatozoa function, thereby reducing men’s fertilizing potential. Differences in PUFA concentrations and ratios among various groups of infertile men, particularly between the teratozoospermic, azoospermic, and oligoasthenoteratozoospermic groups, are likely primarily driven by the markedly reduced sperm count or complete absence of spermatozoa in semen associated with reduced DHA release from the cell membrane. This may explain the lower LA/DHA, ALA/DHA, GLA/DHA, and AA/DHA ratios observed in the teratozoospermic group compared to the azoospermic and oligoasthenoteratozoospermic groups. Also, we observed that variations in other PUFA ratios may result from differences in the concentrations of individual PUFAs across groups.

Despite extensive research on seminal plasma as a source of male infertility biomarkers, the analysis of PUFA profiles in this body fluid as a parameter differentiating fertile and infertile men—considering a broad spectrum of reproductive disorders without stratification into specific patient subgroups–remains largely underexplored area. To the best of our knowledge, the present study is the first to demonstrate the clinical potential of the examined parameters in distinguishing between fertile and infertile men, although Wang et al.^41^ suggested that DHA and AA may serve as potential seminal plasma biomarkers of poor semen quality. It is important to note, however, that due to the limited size of group of fertile men in the present study, further research involving larger cohorts of fertile men is necessary to fully assess the diagnostic potential of PUFAs in male infertility diagnostics. It should also be emphasized that the selection of fertile men must be approached with methodological rigor, as only individuals with proven fertility (who have fathered biological offspring), as in the present study, can serve as a reliable reference point in the context of idiopathic male infertility diagnostics. Among omega-6 PUFAs, LA showed an AUC of 0.674 (95% CI: 0.575–0.773) with a cut-off point of 0.554 μg/mL (Sens. 64.4%, Spec. 68.2%; *p* < 0.001), and GLA yielded an AUC of 0.679 (95% CI: 0.583–0.776) at a cut-off point of 0.039 μg/mL (Sens. 41.2%, Spec. 90.9%; p < 0.001). Of the omega-3 PUFAs, EPA demonstrated the highest discriminative potential with an AUC of 0.709 (95% CI: 0.597–0.821) at a cut-off point of 0.010 μg/mL (Sens. 46.0%, Spec. 90.9%; p < 0.001), while DHA exhibited an AUC of 0.641 (95% CI: 0.545–0.736) with a cut-off point of 3.637 μg/mL (Sens. 32.4%, Spec. 95.5%; p = 0.004). Regarding PUFA ratios, the LA/AA ratio achieved an AUC of 0.697 (95% CI: 0.576–0.817) with a cut-off point of 0.280 (Sens. 62.8%, Spec. 72.7%; p = 0.001). Additionally, the EPA/ALA, EPA/GLA, and EPA/AA ratios recorded AUCs of 0.665 (95% CI: 0.546–0.784; cut-off point: 0.146; Sens. 80.8%, Spec. 50.0%; p = 0.006), 0.659 (95% CI: 0.549–0.770; cut-off point: 0.283; Sens. 50.4%, Spec. 81.8%; p = 0.004), and 0.641 (95% CI: 0.524–0.757; cut-off point: 0.006; Sens. 34.8%, Spec. 86.4%; p = 0.018), respectively. According to the analysis of previous studies considering AUC value interpretation, the clinical utility of these parameters remains difficult to determine with certainty; however, they might be classified as having limited/poor or moderate/acceptable diagnostic value, suggesting a potential discriminatory power, especially due to the fact that the lower bounds of the confidence intervals in each case exceeded 0.50, with *p* < 0.05 [62]. Existing studies predominantly focus on comparisons between specific patient subgroups, which restricts the ability to draw broader conclusions. Moreover, a major challenge in such research lies in the selection of an appropriate control group. Some authors assume that normozoospermic men are fertile, despite the growing evidence indicating that normal semen parameters do not always correlate with actual fertilization capacity^12,13^. An additional challenge is the revision of diagnostic guidelines for male infertility, as most previous studies have classified men based on earlier reference ranges, which were updated in 2021^1^. Furthermore, information on PUFA ratios in seminal plasma is scarce. Correnti et al.^49^ demonstrated that ALA levels in seminal plasma were higher in infertile men compared to their fertile counterparts. Although the study group included patients with various sperm abnormalities (divided according to WHO 2010 criteria), the sample size was relatively small, and the reference group consisted of normozoospermic men, whose fertility was not explicitly confirmed, considering that these individuals were also patients of an infertility clinic. In our study, no significant differences in seminal plasma ALA concentrations were observed between fertile and infertile men. This discrepancy may stem, among other factors, from differences in the classification of men into appropriate study groups as well as the applied methodology, as the authors employed an alternative lipid extraction protocol (methanol:acetonitrile:water) and utilized ultra-high-pressure liquid chromatography with mass spectrometry (UHPLC-MS)^49^. Similar to the present study, Filipcikova et al.^52^ assigned fertile men with children to the control group. However, the classification of other participants was based on the 1999 WHO criteria. The infertile group was subdivided into normozoospermic men and a mixed group, which included oligozoospermic, asthenozoospermic, and oligoasthenozoospermic patients. The mixed group exhibited higher AA levels (55.9 (48.4–62.5) nmol/μg of protein) and a higher AA/DHA ratio (89.8 (33.8–139.3)), along with a lower DHA concentration (0.6 (0.5–1.5) nmol/μg of protein) than fertile group (AA: 35.5 (32.1–36.9), DHA: 0.5 (2.2–2.9), AA/DHA: 14.1 (12.9–16.1)). In contrast, the normozoospermic group differed from the fertile men only in terms of an elevated AA/DHA ratio (52.2 (20.2–61.8))^52^. Comparing our findings with those by Filipcikova et al.^52^ is challenging due to differences in the classification of infertile men, the heterogeneity of the studied groups, and the distinct approach to expressing PUFA concentrations in seminal plasma. Nevertheless, in the context of comparisons between normozoospermic infertile patients and fertile men, our findings align with those of Filipcikova et al.^52^, as we also did not observe significant differences in AA and DHA concentrations between these groups. In contrast, Obrona et al.^55^, using the same patient classification and methodological approach as Filipcikova et al.^52^, observed that in the group of normozoospermic infertile patients, DHA levels were lower, while AA concentrations and the AA/DHA ratio were higher compared to fertile men. In both studies, the investigated groups were relatively small, which may have influenced the obtained results and drawn conclusions. Several authors compared asthenozoospermic men with normozoospermic individuals^43,46,47^. However, among them, only Tang et al.^43^ used seminal plasma from sperm donors as the control group, which enhanced its credibility as representative of fertile men, although it was not entirely equivalent. The authors classified participants according to the 2010 WHO criteria and utilized a chloroform:methanol extraction method followed by GC-FID for PUFAs analysis. Although they reported lower DHA and higher AA concentrations in the seminal plasma of men with asthenozoospermia compared to the sperm donors’ group, these differences did not reach statistical significance, which is consistent with our findings. Yu et al.^46^ and Conquer et al.^47^ conducted comparative studies on asthenozoospermic and normozoospermic patients based on the 1999 WHO criteria. Yu et al.^46^ demonstrated that AA levels in seminal plasma were higher in asthenozoospermic men (17.170 ± 4.073 mg/mL vs. 14.274 ± 3.622 mg/mL), whereas Conquer et al.^47^ observed that not only AA (3.3% vs. 3.1%) but also ALA (0.19% vs. 0.19%) and LA (2.5% vs. 2.3%) content did not show significant differences between groups, while DHA content was higher in the seminal plasma of asthenozoospermic men (3.7% vs. 3.0% for normozoospermic). Discrepancies in the presented findings may stem from methodological differences, as Yu et al. employed HPLC–ESI–MS/MS^46^, whereas Conquer et al. utilized GC-FID^47^. Our results are inconsistent with those of Yu et al.^46^ regarding AA and with those of Conquer et al.^47^ concerning DHA, which may be attributed not only to differences in analytical techniques but also to variations in the ethnic background of the studied populations. In both aforementioned studies, the participants were of Chinese origin, where PUFA intake differs from that in European countries, suggesting that dietary factors may influence the observed results^60^. In summary, interpretation of the findings is challenging due to several factors, including the heterogeneous classification criteria for fertile and infertile men, the limited sample sizes, variations in PUFA extraction and examination methods, and differences in how results are expressed. Additionally, environmental factors such as diet may influence PUFA metabolism, contributing to variability across studies. An important consideration is that most studies do not account for the full spectrum of relevant PUFAs in their analyses, and their ratios are rarely provided, even though the findings presented in our study demonstrate their application potential in differentiating subgroups of infertile patients. Nevertheless, changes in the classification of infertile men, particularly those introduced between 2010 and 2021, appear to have a limited impact on the overall conclusions. In our study, classifying patients according to the 2010 WHO criteria did not yield significant differences compared to the 2021 classification, suggesting that these revisions do not substantially alter the interpretation of PUFA’s role in male infertility (Table S5, Supplementary Materials).

The analysis of correlations between the concentrations of examined parameters and standard semen parameters revealed that only DHA exhibited moderate positive correlations with sperm concentrations, total sperm count in the ejaculate, and the concentrations of morphologically abnormal sperm. Notably, despite the observed higher concentrations of DHA in the seminal plasma of men in the teratozoospermic group–suggesting increased PUFA release from the spermatozoa membranes due to their damage–no correlation was found between DHA concentrations and the percentage of spermatozoa with normal morphology. Following an analysis of the possible causes of this observation, an additional parameter was introduced: concentration of morphologically abnormal sperm (calculated based on sperm concentration and the percentage of sperm with normal morphology). It allows us to confirm that DHA concentration increased with the concentration of damaged sperm in the semen. Therefore, we believe that the analysis of mechanisms underlying male reproductive potential decline should also consider the biological role of this parameter, as it appears to better reflect the possible causes of changes in seminal plasma composition due to sperm damage, rather than the percentage of morphologically normal gametes. Our results are consistent with those obtained by Huang et al.^56^, who found a positive correlation between DHA level in seminal plasma and sperm concentration as well as total sperm count. Our results also align with correlations between DHA levels and semen parameters observed by Martinez-Soto et al.^24^ who reported a moderate positive correlation of DHA concentrations with total sperm count in ejaculate (R = 0.52) and its weak positive correlations with sperm motility (R = 0.19) and morphology (R = 0.22). Furthermore, Wang et al.^41^ demonstrated positive correlations between DHA levels in seminal plasma and total sperm count, concentration, motility, and normal sperm morphology among Chinese men, including those with proven fertility. Comparable results for DHA were reported by Safarinejad et al.^48^, who focused exclusively on men with oligoasthenoteratozoospermia. The authors found significant positive correlations between DHA levels and sperm concentrations (R = 0.62), total sperm count (R = 0.61), motility (R = 0.60), and morphology (R = 0.55). Additionally, they observed similar correlations for EPA (R = 0.56, R = 0.57, R = 0.54, and R = 0.48, respectively)^48^. Although we also identified associations between DHA levels and total sperm motility as well as their normal morphology, the former was weak, and the latter was not significant. In contrast, we did not observe significant correlations for EPA. Other studies have reported divergent findings. For example, Gao et al.^50^ and Attaman et al.^54^ did not find significant correlations between seminal plasma DHA levels and semen parameters. Abdollahzadeh et al.^53^ reported only a correlation between DHA levels and sperm motility (R = 0.54). However, our results align partially with those obtained by Gao et al.^50^ and Abdollahzadeh et al.^53^, who did not demonstrate correlations between ALA or EPA levels and semen parameters. Moreover, similarly to Martinez-Soto et al.^24^, we did not observe correlations between semen parameters and LA or AA levels, while in the case of EPA, we reported only a very weak correlation with total sperm count. Our observations are also consistent with those of Attaman et al.^54^, who did not identify significant correlations between seminal plasma levels of ALA, LA, and AA and semen parameters. The differences observed in the correlations may stem from several factors, including the heterogeneous characteristics of the study populations, the methodologies employed to quantify PUFA levels in seminal plasma, as well as variations in demographic, dietary, lifestyle factors, and environmental exposures among the study participants. To the best of our knowledge, correlations between PUFA ratios and semen parameters have not been reported in the available literature. Although the observed moderate negative correlations between the LA/DHA, ALA/DHA, GLA/DHA, AA/DHA, and EPA/DHA ratios and semen parameters (especially sperm concentration, count per ejaculate and concentration of morphologically abnormal sperm) are likely a direct result of the correlation between DHA levels and sperm concentrations, it is important to note that changes in the PUFA content in seminal plasma may be due to factors other than the release of these fatty acids from spermatozoa membranes and an imbalance in PUFAs intake and/or metabolism may also negatively impact semen quality.

Based on the above-mentioned findings, several preliminary yet real recommendations can be formulated for future clinical and research practice. First, selected PUFAs and their ratios—particularly EPA and EPA-related indices (EPA/GLA, EPA/ALA)—should be further examined and considered as potential components of an extended diagnostic panel in the assessment of male infertility. Pending validation in larger, more balanced cohorts, these biomarkers may aid in refining patient classification and tailoring infertility treatment strategies. Second, the observed associations between PUFA levels and sperm morphology abnormalities in teratozoospermic men, suggest that analysis of seminal plasma lipid composition could complement conventional semen analysis in clinical settings, providing insight into the underlying sperm malformations/molecular damage. In our opinion, the concentration of morphologically abnormal sperm should be incorporated as parameter into standard laboratory protocols. Its implementation will enhance the diagnostic precision of semen evaluations. Third, the introduction of the optimized GC–MS/MS analytical method for the quantification of seminal plasma PUFAs into clinical practice offers a promising tool for precise and reliable assessment of these biomarkers. High sensitivity and specificity of this method can enhance diagnostic accuracy and support personalized therapeutic decisions, once validated in larger cohorts.

## Strengths, limitations and future perspectives

The findings of the present study indicate that seminal plasma serves not only as a valuable source of potential biomarkers for male infertility but also provides insights into the underlying mechanisms contributing to reduced fertility and their potential reflection in the composition of seminal plasma. Targeted interventions addressing these mechanisms could represent a significant advancement in the development of therapeutic and preventive strategies. However, due to the observational nature of this study, it is not possible to conclusively determine which PUFAs may serve as critical biomarker for male infertility, despite the promising differences observed. Therefore, further research involving larger cohorts of both infertile patients and proven fertile men is warranted. One of the key strengths of the present study lies in its methodological innovations. The optimization of the extraction method for seminal plasma PUFAs, combined with the novel application of GC–MS/MS for precise quantification, opens new directions not only for infertility diagnostics but also for biomarkers identification in other medical conditions. Nevertheless, further validation of these methods in larger cohorts is needed. The observed differences in the concentrations of individual PUFAs and PUFA ratios between fertile and infertile men—both in the entire study population and in subgroups stratified by specific infertility disorders, including idiopathic infertility—highlight the diagnostic potential of these parameters, which underscores the need for further research in this field. Another strength of the present study is the relatively large and carefully selected group of infertile patients, encompassing a broad spectrum of sperm-related abnormalities in concentration, motility, and morphology. Furthermore, the inclusion of a control group composed of men with proven fertility enhances the scientific and clinical relevance of the findings, although its size is limited. This aspect is particularly important given that previous studies often utilized normozoospermic men as controls without excluding idiopathic/unexplained infertility. Future research should incorporate a wider range of infertility-related disorders to provide a more comprehensive representation of the actual variations and interrelations across the entire infertile male population. Of particular significance is the inclusion of men with teratozoospermia, as our findings suggest that seminal plasma PUFA levels may reflect structural sperm damage, contributing to abnormal morphology observed in routine semen analysis. Additionally, the potential influence of environmental and dietary factors on the molecular composition of seminal plasma, including PUFA concentrations and ratios, should not be overlooked. These factors may ultimately affect semen parameters and modulate male reproductive potential. Therefore, future studies should not only examine the impact of such factors on fertility but also integrate them into the interpretation of seminal plasma composition data. It is conceivable that the findings from such research could provide an impetus for dietary modifications in the context of male fertility. The observed differences between azoospermic men and other groups suggest that seminal plasma analysis could serve as a potential non-invasive alternative to testicular biopsy in cases of azoospermia, however, this should be considered a hypothesis that goes beyond the design of the present study and serves primarily to indicate a potential direction for future research. Further research is required to determine whether the identified alterations in PUFAs content are directly associated with the presence or absence of spermatozoa in the epididymis and testes. A deeper understanding of these associations could enable the prediction of spermatozoa retrieval success prior to the necessity of invasive biopsy procedures. Additionally, the correlations between PUFA concentrations/ratios and conventional semen parameters provide valuable insights into both the mechanisms responsible for seminal plasma PUFA alterations, such as sperm membrane damage, and their impact on male reproductive potential. This knowledge could be pivotal in the development of personalized therapeutic and preventive strategies. However, further investigations are required to elucidate the underlying causes of these changes. Future research should focus on defining the role of oxidative stress in alterations of PUFA composition in seminal plasma and analyzing PUFA metabolites and their potential effects on spermatozoa functionality. Another noteworthy contribution of our study is the introduction of a novel parameter—concentration of morphologically abnormal sperm—which appears to better reflect actual correlations than the percentage of morphologically normal sperm. Despite the relatively frequent updates to diagnostic criteria for male infertility, the modification introduced by the WHO in 2021 does not appear to affect the conclusions drawn from previous analyses based on the 2010 classification regarding PUFAs. Nevertheless, it should be considered when comparing past and current findings.

Despite the numerous significant conclusions derived from the present study, certain limitations must be indicated. One of the most important limitations of the present study lies in the substantial imbalance between the number of fertile controls and infertile patients, resulting in a skewed group ratio that may compromise the statistical robustness of the findings when infertile patients are analyzed together, without differentiating into subgroups depending on semen analysis results. Although the control group was rigorously selected to ensure confirmed fertility, its relatively small size limits the statistical power, increases the risk of sampling bias, and may affect the precision of estimated effect sizes. Consequently, the observed associations, while promising, should be interpreted with caution. We fully acknowledge that the imbalance in group sizes restricts the generalizability and strength of our conclusions, particularly regarding the diagnostic potential of PUFAs. Future studies involving larger and more evenly distributed cohorts will be essential to validate our findings and strengthen the clinical relevance of PUFA-related biomarkers in male infertility diagnostics. Moreover, the lack of detailed data on environmental factors, diet and supplementation that may modulate seminal plasma composition and semen parameters constrains the full interpretation of the observed correlations, which remain largely hypothetical at this stage. Additionally, although the clinical utility of the investigated parameters in differential diagnosis is proposed, the presented results demonstrate only moderate or acceptable clinical applicability, which may be either confirmed or refuted in future studies involving larger cohorts of men.

## Conclusions

The present observational study suggests PUFA profiles may differ among infertility subgroups as well as when compared to fertile men. Thanks to the optimization of the extraction method from seminal plasma, it is possible to accurately quantify their content using lower sample volumes and a reduced analysis time. Given the current limitations in male infertility diagnostics, our work introduces new possibilities also by successfully applying, for the first time, GC–MS/MS in the analysis of human seminal plasma PUFAs. Comparative analysis revealed significant differences in nine out of twenty-one examined parameters between infertile patients and a control group of proven fertility. These differences encompassed the concentrations of LA, GLA, EPA, and DHA, as well as the ratios of ALA/LA, LA/AA, EPA/ALA, EPA/GLA, and EPA/AA. Among these, five parameters (LA, GLA, LA/AA, EPA/ALA, and EPA/GLA) exhibited limited, while one (EPA) demonstrated moderate diagnostic value. Notably, we also observed differences among subgroups of infertile patients, including those with idiopathic infertility. The teratozoospermic group displayed the most pronounced alterations, characterized by generally higher PUFA concentrations (particularly DHA), whereas the azoospermic group exhibited lower PUFA levels. The observed trend suggests PUFA release from sperm membranes because of their structural damage. Additionally, both the teratozoospermic and azoospermic groups significantly differed from other subgroups in seminal plasma PUFA ratios, particularly LA/DHA, ALA/DHA, GLA/DHA, and AA/DHA, further confirming the potential discriminatory power of these parameters. However, due to the relatively small control group of fertile men, these promising findings should be interpreted with caution, and require confirmation in larger, more statistically balanced cohorts. Correlations analysis revealed moderate associations between selected PUFA-related parameters (DHA, LA/DHA, ALA/DHA, GLA/DHA, AA/DHA, EPA/DHA) and semen parameters, particularly sperm concentration and the concentration of morphologically abnormal sperm. Notably, the latter, a novel parameter introduced in our study, appears to better reflect spermatozoa damage and its relationship with PUFA composition and function than the conventional percentage of morphologically normal spermatozoa reported in routine semen analysis. Moreover, our findings indicate that the 2021 WHO criteria revision for male infertility classification did not substantially impact conclusions derived from the previous studies in which WHO 2010 classification was used for infertile groups differentiation. Nevertheless, this factor should be carefully considered when interpreting past research findings.

**Fig. 5:**
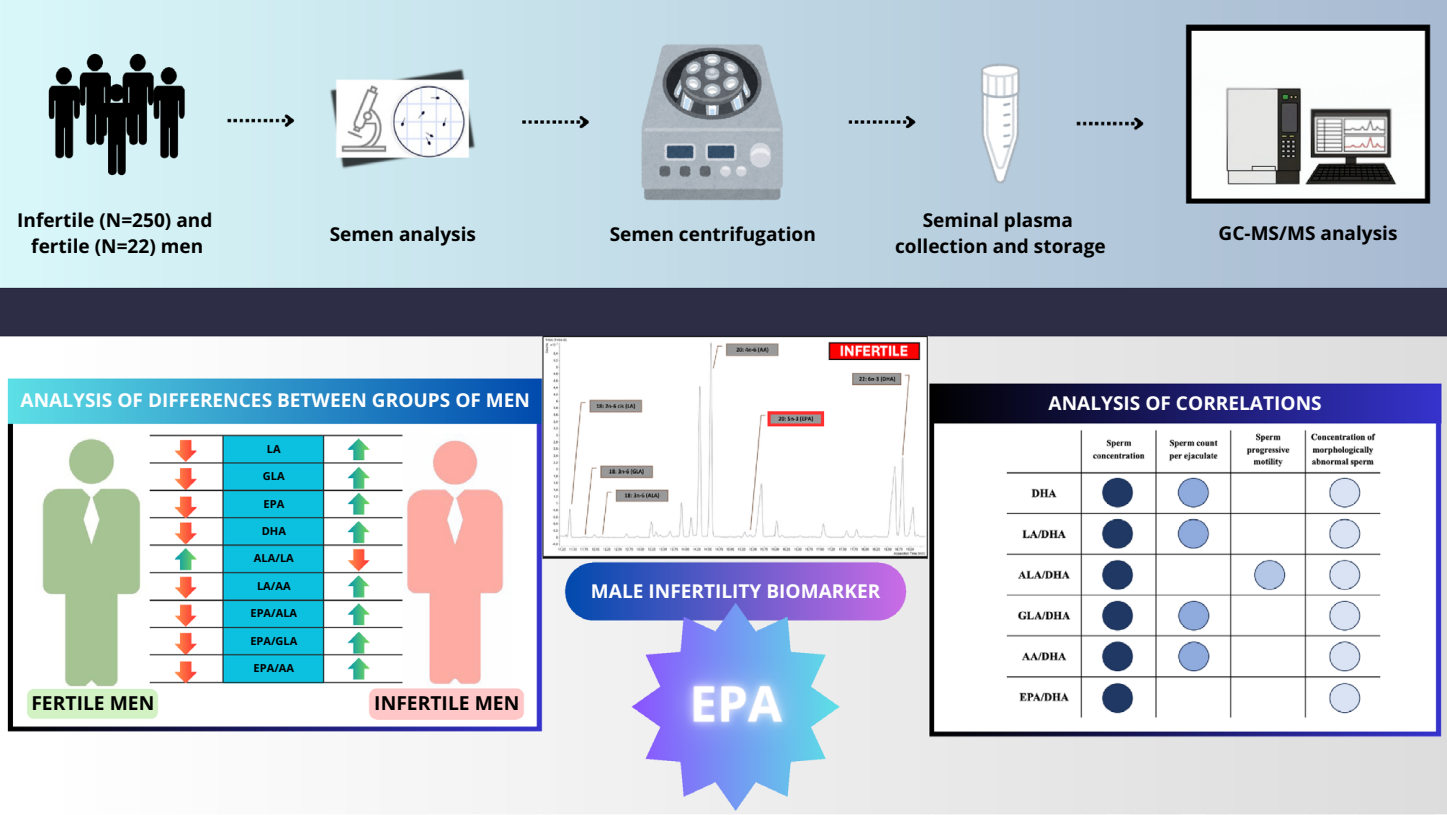
Summary of the main findings of the study. AA: arachidonic acid, ALA: α-linolenic acid, DHA: docosahexaenoic acid, EPA: eicosapentaenoic acid, GC–MS/MS: gas chromatography–tandem mass spectrometry, GLA: γ-linolenic acid, LA: linoleic acid.

In summary, the present study shows preliminary insights into the probable associations between selected PUFAs expression in seminal plasma and some semen abnormalities resulted in male infertility. We are convinced that the identification and complete understanding of mechanisms provided to this disease may pave the way for the development of new effective therapeutic and preventive interventions. Even though this is currently only a suggestion that requires verification in further, more extensive research, we want to believe that the first step on this path has already been taken. A summary of the main findings of the present study is provided in Fig. 5.

## Supplementary Information

Supplementary Information.

## Data Availability

All data generated or analyzed during this study are included in this published article (and its Supplementary Information files).

## Abbreviations

A: Asthenozoospermic group
AA: Arachidonic acid
ALA: α-Linolenic acid
AT: Asthenoteratozoospermic group
Azoo: Azoospermic group
BHT: 2,6-Di-*tert*-butyl-4-methylphenol
DHA: Docosahexaenoic acid
EPA: Eicosapentaenoic acid
F: Fertile group
FAME(s): Fatty acid methyl ester(s)
FID: Flame ionization detection
GC: Gas chromatography
GC–MS/MS: Gas chromatography–tandem mass spectrometry
GLA: γ-Linolenic acid
LA: Linoleic acid
Me: Median
MRM: Multiple reaction monitoring
MS: Mass spectrometry
MS/MS: Tandem mass spectrometry
NI: Normozoospermic infertile group
OAT: Oligoasthenoteratozoospermic group
OT: Oligoteratozoospermic group
PUFA(s): Polyunsaturated fatty acid(s)
QC: Quality control
Q1–Q3: Interquartile range
ROC: Receiver operating characteristic
T: Teratozoospermic group
UHPLC-MS: Ultra-high-pressure liquid chromatography with mass spectrometry
WHO: World Health Organization
