# Supplementary material for: GC–MS/MS analysis of seminal plasma PUFAs in distinct subgroups of infertile men: diagnostic potential and insight into mechanisms of male infertility
Source: Sci Rep. 2025 Oct 3;15:34577. doi: 10.1038/s41598-025-18044-4 (PMC12494725; doi:10.1038/s41598-025-18044-4)
Supplement: Supplementary file 1 — Supplementary Information. [file 41598_2025_18044_MOESM1_ESM.docx]

Supplementary Materials for

**GC-MS/MS Analysis of Seminal Plasma PUFAs in Distinct Subgroups of Infertile Men – Diagnostic Potential and Insight into Mechanisms of Male Infertility**

Kamil Rodak*, Magdalena Grajzer, Izabela Kokot, Ricardo Faundez, Iwona Gilowska, Anna Prescha, Ewa Maria Kratz* (*Co-corresponding authors)

Correspondence to: [krodak98@icloud.com](mailto:krodak98@icloud.com)

[ewa.kratz@umw.edu.pl](mailto:ewa.kratz@umw.edu.pl)

**Table S1** Comparison of the PUFA concentrations/ratios in seminal plasma between the fertile and infertile men (insignificant differences, *p* > 0.05)

| **PUFA** | **PUFA concentration [μg/mL]**  **Me**  **(Q1-Q3)** | | ***p*-value** |
| --- | --- | --- | --- |
|  | **Fertile (n = 22)** | **Infertile (n = 250)** |  |
| **ALA**  **(18:3 n-3)** | 0.032  (0.023–0.048) | 0.037  (0.025–0.056) | *p* = 0.392 |
| **AA**  **(20:4 n-6)** | 2.193  (1.480–3.155) | 2.478  (1.504–4.297) | *p* = 0.221 |
| **GLA/LA** | 0.056  (0.038–0.075) | 0.046  (0.032–0.068) | *p* = 0.093 |
| **EPA/LA** | 0.012  (0.008–0.019) | 0.013  (0.009–0.023) | *p* = 0.420 |
| **LA/DHA** | 0.305  (0.233–0.458) | 0.340  (0.271–0.451) | *p* = 0.443 |
| **ALA/GLA** | 1.174  (0.935–1.620) | 1.123  (0.793–1.575) | *p* = 0.397 |
| **ALA/AA** | 0.016  (0.012–0.021) | 0.016  (0.009–0.025) | *p* = 0.658 |
| **ALA/DHA** | 0.023  (0.013–0.036) | 0.016  (0.009–0.031) | *p* = 0.166 |
| **GLA/AA** | 0.013  (0.009–0.017) | 0.014  (0.009–0.022) | *p* = 0.744 |
| **GLA/DHA** | 0.018  (0.012–0.027) | 0.016  (0.010–0.024) | *p* = 0.355 |
| **AA/DHA** | 1.330  (0.944–1.636) | 1.115  (0.871–1.465) | *p* = 0.116 |
| **EPA/DHA** | 0.004  (0.002–0.007) | 0.005  (0.003–0.008) | *p* = 0.270 |

The U Mann-Whitney test was used to assess the differences between groups. A two-tailed *p*-value of less than 0.05 was considered significant. AA – arachidonic acid, ALA – α-linolenic acid, DHA – docosahexaenoic acid, EPA – eicosapentaenoic acid, GLA – γ-linolenic acid, LA – linoleic acid, Me – median, PUFA – polyunsaturated fatty acid, Q1-Q3 – interquartile range.

**Table S2** Summary of the results of ROC curves analysis for parameters with AUC < 0.65

|  | AUC | AUC - 95% Confidence Interval | Cut-off point | Sensitivity [%] | Specificity [%] | *p*-value |
| --- | --- | --- | --- | --- | --- | --- |
| **ALA** | 0.558 | 0.440–0.676 | 0.037 | 50.8 | 63.6 | 0.337 |
| **AA** | 0.579 | 0.447–0.680 | 4.427 | 23.6 | 100.00 | 0.131 |
| **DHA** | 0.641 | 0.545–0.736 | 3.637 | 32.4 | 95.5 | 0.004 |
| **ALA/LA** | 0.631 | 0.528–0.735 | 0.048 | 46.4 | 86.4 | 0.012 |
| **GLA/LA** | 0.608 | 0.498–0.717 | 0.054 | 67.2 | 50.0 | 0.054 |
| **EPA/LA** | 0.559 | 0.448–0.669 | 0.026 | 21.2 | 95.5 | 0.301 |
| **LA/DHA** | 0.549 | 0.411–0.687 | 0.260 | 78.0 | 45.5 | 0.488 |
| **ALA/GLA** | 0.445 | 0.334–0.557 | 2.027 | 14.0 | 90.9 | 0.336 |
| **ALA/AA** | 0.473 | 0.364–0.583 | 0.038 | 13.6 | 95.5 | 0.635 |
| **ALA/DHA** | 0.590 | 0.479–0.702 | 0.016 | 50.4 | 68.2 | 0.113 |
| **GLA/AA** | 0.525 | 0.406–0.643 | 0.018 | 36.4 | 77.3 | 0.683 |
| **GLA/DHA** | 0.440 | 0.328–0.552 | 0.051 | 6.8 | 100 | 0.297 |
| **EPA/AA** | 0.641 | 0.524–0.757 | 0.006 | 34.8 | 86.4 | 0.018 |
| **AA/DHA** | 0.601 | 0.482–0.721 | 1.177 | 57.6 | 63.6 | 0.096 |
| **EPA/DHA** | 0.569 | 0.445–0.693 | 0.005 | 54.0 | 63.6 | 0.275 |

Data are given as AUC with 95% confidence interval. A *p*-value of less than 0.05 was considered significant. AA – arachidonic acid, ALA – α-linolenic acid, DHA – docosahexaenoic acid, EPA – eicosapentaenoic acid, GLA – γ-linolenic acid, LA – linoleic acid.

**Table S3** Comparison of the PUFA ratios in seminal plasma between the examined groups of men (insignificant differences, *p* > 0.05)

| **Group**  **Ratio** | | **T** | **A** | **Azoo** | **NI** | **AT** | **OT** | **OAT** | **F** |
| --- | --- | --- | --- | --- | --- | --- | --- | --- | --- |
| **Me**  **(Q1-Q3)** | **GLA/LA** | 0.041  (0.029–0.055) | 0.039  (0.032–0.071) | 0.073  (0.045–0.094) | 0.044  (0.032–0.064) | 0.047  (0.030–0.073) | 0.045  (0.030–0.055) | 0.048  (0.039–0.064) | 0.056  (0.038–0.075) |
|  | **LA/AA** | 0.321  (0.269–0.371) | 0.369  (0.223–0.457) | 0.293  (0.238–0.379) | 0.329  (0.272–0.376) | 0.295  (0.245–0.361) | 0.305  (0.269–0.345) | 0.293  (0.248–0.408) | 0.248  (0.199–0.306) |
|  | **EPA/LA** | 0.018  (0.010–0.027) | 0.010  (0.006–0.012) | 0.014  (0.012–0.016) | 0.010  (0.006–0.016) | 0.011  (0.007–0.017) | 0.010  (0.009–0.023) | 0.018  (0.010–0.029) | 0.012  (0.008–0.019) |
|  | **ALA/AA** | 0.011  (0.006–0.019) | 0.019  (0.014–0.029) | 0.021  (0.016–0.042) | 0.013  (0.008–0.030) | 0.017  (0.012–0.031) | 0.015  (0.009–0.022) | 0.016  (0.010–0.026) | 0.016  (0.012–0.021) |
|  | **GLA/AA** | 0.012  (0.009–0.021) | 0.018  (0.011–0.020) | 0.023  (0.013–0.031) | 0.012  (0.009–0.021) | 0.014  (0.009–0.022) | 0.013  (0.008–0.017) | 0.016  (0.012–0.021) | 0.013  (0.009–0.017) |
|  | **EPA/AA** | 0.005  (0.003–0.010) | 0.003  (0.002–0.005) | 0.004  (0.003–0.006) | 0.003  (0.002–0.005) | 0.003  (0.002–0.005) | 0.003  (0.002–0.007) | 0.005  (0.003–0.010) | 0.003  (0.002–0.004) |

The ANOVA followed by Tukey’s test was used to assess the differences between groups. A two-tailed *p*-value of less than 0.05 was considered significant. A – asthenozoospermic group, AA – arachidonic acid, ALA – α-linolenic acid, Azoo – azoospermic group, AT – asthenoteratozoospermic group, EPA – eicosapentaenoic acid, F – fertile group, GLA – γ-linolenic acid, LA – linoleic acid, Me – median, NI – normozoospermic infertile group, OAT – oligoasthenoteratozoospermic group, OT – oligoteratozoospermic group, PUFA – polyunsaturated fatty acid, Q1-Q3 – interquartile range, T – teratozoospermic group.

**Table S4** Correlations between PUFA concentrations/ratios and semen parameters (weak and/or insignificant)

| **Parameter**  **PUFA** | **Sperm concentration**  **[×10^6^/mL]** | | **Sperm count per ejaculate**  **[×10^6^]** | | **Sperm viability**  **[%]** | | **Sperm total motility**  **[%]** | | **Sperm progressive motility**  **[%]** | | **Sperm normal morphology**  **[%]** | | **Concentration of morphologically abnormal sperm  [×10^6^/mL]** | |
| --- | --- | --- | --- | --- | --- | --- | --- | --- | --- | --- | --- | --- | --- | --- |
|  | **R** | ***p*** | **R** | ***p*** | **R** | ***p*** | **R** | ***p*** | **R** | ***p*** | **R** | ***p*** | **R** | ***p*** |
| **LA (18:2 n-6 cis)** | 0.23 | < 0.001 | 0.21 | < 0.001 | 0.12 | 0.050 | 0.21 | < 0.001 | 0.25 | < 0.001 | 0.13 | 0.047 | 0.23 | < 0.001 |
| **ALA**  **(18:3 n-3)** | 0.03 | 0.059 | 0.03 | 0.581 | -0.03 | 0.635 | -0.07 | 0.285 | -0.13 | 0.038 | 0.01 | 0.869 | 0.04 | 0.557 |
| **GLA**  **(18:3 n-6)** | 0.12 | 0.045 | 0.13 | 0.028 | 0.09 | 0.166 | 0.15 | 0.011 | 0.16 | 0.007 | -0.02 | 0.792 | 0.12 | 0.039 |
| **AA**  **(20:4 n-6)** | 0.21 | < 0.001 | 0.18 | 0.004 | 0.15 | 0.019 | 0.20 | < 0.001 | 0.22 | < 0.001 | 0.02 | 0.703 | 0.21 | < 0.001 |
| **EPA**  **(20:5 n-3)** | 0.06 | 0.360 | 0.13 | 0.033 | 0.04 | 0.524 | 0.17 | 0.005 | 0.25 | < 0.001 | -0.12 | 0.057 | 0.06 | 0.309 |
| **ALA/LA** | -0.18 | 0.003 | -0.15 | 0.014 | -0.10 | 0.118 | -0.23 | < 0.001 | -0.34 | < 0.001 | -0.10 | 0.099 | -0.18 | 0.003 |
| **GLA/LA** | -0.22 | < 0.001 | -0.17 | 0.005 | -0.09 | 0.148 | -0.16 | 0.008 | -0.20 | 0.001 | -0.08 | 0.187 | -0.22 | < 0.001 |
| **LA/AA** | 0.05 | 0.396 | 0.09 | 0.126 | -0.06 | 0.383 | 0.02 | 0.738 | 0.06 | 0.346 | 0.08 | 0.206 | 0.06 | 0.357 |
| **EPA/LA** | -0.17 | 0.005 | -0.07 | 0.237 | -0.04 | 0.481 | -0.01 | 0.835 | 0.04 | 0.563 | -0.10 | 0.111 | -0.17 | 0.006 |
| **ALA/GLA** | -0.04 | 0.547 | -0.05 | 0.452 | -0.08 | 0.181 | -0.16 | 0.008 | -0.23 | < 0.001 | -0.04 | 0.527 | -0.04 | 0.559 |
| **ALA/AA** | -0.14 | 0.021 | -0.10 | 0.098 | -0.11 | 0.075 | -0.20 | 0.001 | -0.29 | < 0.001 | -0.05 | 0.436 | -0.14 | 0.024 |
| **EPA/ALA** | 0.01 | 0.921 | 0.06 | 0.355 | 0.04 | 0.494 | 0.19 | 0.002 | 0.30 | < 0.001 | -0.04 | 0.556 | 0.01 | 0.886 |
| **GLA/AA** | -0.19 | 0.002 | -0.12 | 0.049 | -0.12 | 0.063 | -0.15 | 0.013 | -0.18 | 0.003 | -0.02 | 0.742 | -0.19 | 0.002 |
| **EPA/GLA** | -0.02 | 0.795 | 0.05 | 0.372 | 0.01 | 0.990 | 0.11 | 0.063 | 0.20 | 0.001 | -0.07 | 0.287 | -0.01 | 0.853 |
| **EPA/AA** | -0.14 | 0.018 | -0.04 | 0.514 | -0.08 | 0.233 | -0.02 | 0.699 | 0.02 | 0.683 | -0.07 | 0.305 | -0.14 | 0.023 |

Spearman’s rank test was used to assess the correlations between analyzed parameters, and a *p*-value of less than 0.05 was considered significant (red font). AA – arachidonic acid, ALA – α-linolenic acid, DHA – docosahexaenoic acid, EPA – eicosapentaenoic acid, GLA – γ-linolenic acid, LA – linoleic acid, PUFA – polyunsaturated fatty acid.

| **PUFA**  **Group** | **LA (18:2 n-6 *cis*)**  **[μg/mL]** | **ALA**  **(18:3 n-3)**  **[μg/mL]** | **GLA**  **(18:3 n-6)**  **[μg/mL]** | **AA**  **(20:4 n-6)**  **[μg/mL]** | **EPA**  **(20:5 n-3)**  **[μg/mL]** | **DHA**  **(22:6 n-3)**  **[μg/mL]** |
| --- | --- | --- | --- | --- | --- | --- |
|  | **Me  (Q1-Q3)** | | | | | |
| **T** | **1.071 ^a,b^**  **(0.724–1.750)** | 0.030  (0.019–0.065) | **0.042 ^c^**  **(0.032–0.052)** | **3.382 ^d^**  **(2.061–5.653)** | **0.017 ^e,f,g^**  **(0.011–0.030)** | **4.408 ^h,i,j,k,l,m^**  **(2.528–5.709)** |
| **A** | 0.813  (0.487–1.173) | 0.044  (0.036–0.055) | 0.034  (0.030–0.040) | 2.094  (1.592–3.987) | 0.007  (0.005–0.009) | 2.317  (1.503–3.531) |
| **Azoo** | 0.464  (0.327–0.639) | 0.039  (0.030–0.044) | 0.031  (0.023–0.040) | 1.445  (0.972–2.408) | 0.007  (0.006–0.008) | 0.799  (0.502–1.093) |
| **NI** | 0.549  (0.420–1.076) | 0.023  (0.017–0.037) | 0.024  (0.021–0.034) | 2.283  (1.620–3.341) | 0.006  (0.004–0.008) | 1.645  (1.280–3.473) |
| **AT** | 0.681  (0.431–1.185) | 0.044  (0.030–0.064) | 0.032  (0.025–0.040) | 2.154  (1.289–4.378) | 0.008  (0.006–0.010) | 2.365  (1.238–4.389) |
| **OT** | 0.645  (0.469–1.709) | 0.040  (0.027–0.048) | 0.030  (0.025–0.040) | 3.238  (1.573–5.150) | 0.009  (0.005–0.016) | 1.897  (1.660–2.849) |
| **OAT** | 0.727  (0.380–1.142) | 0.036  (0.029–0.050) | 0.037  (0.026–0.045) | 2.419  (1.576–3.786) | 0.010  (0.008–0.019) | 1.847  (1.181–2.482) |
| **F** | 0.493  (0.335–0.838) | 0.032  (0.023–0.048) | 0.029  (0.021–0.033) | 2.193  (1.480–3.155) | 0.007  (0.004–0.008) | 1.519  (0.083–2.508) |

**Table S5** PUFA concentrations in groups of examined men classified according to the 2010 WHO criteria

^a^ T vs. Azoo; ^b^ T vs. F; ^c^ T vs. F; ^d^ T vs. Azoo; ^e^ T vs Azoo; ^f^ T vs. AT; ^g^ T vs. F; ^h^ T vs. Azoo; ^i^ T vs. NI; ^j^ T vs. AT; ^k^ T vs. OT; ^l^ T vs. OAT; ^m^ T vs. F. ^a-m^ p < 0.05.
AA – arachidonic acid, ALA – α-linolenic acid, Azoo – azoospermic group, AT – asthenoteratozoospermic group, DHA – docosahexaenoic acid, EPA – eicosapentaenoic acid, F – fertile group, GLA – γ-linolenic acid, LA – linoleic acid, Me – median, NI – normozoospermic infertile group, OAT – oligoasthenoteratozoospermic group, OT – oligoteratozoospermic group, PUFA – polyunsaturated fatty acid, Q1-Q3 – interquartile range, T – teratozoospermic group. Values with statistical significance are indicated in bold font.
